# Detecting minimum energy states and multi-stability in nonlocal advection–diffusion models for interacting species

**DOI:** 10.1007/s00285-022-01824-1

**Published:** 2022-10-20

**Authors:** Valeria Giunta, Thomas Hillen, Mark A. Lewis, Jonathan R. Potts

**Affiliations:** 1 School of Mathematics and Statistics, University of Sheffield, Hicks Building, Hounsfield Road, Sheffield, S3 7RH UK; 2 Department of Mathematical and Statistical Sciences, University of Alberta, Edmonton, AB T6G 2G1 Canada; 3 Department of Mathematics and Statistics and Department of Biology, University of Victoria, PO Box 1700 Station CSC, Victoria, BC Canada

**Keywords:** Animal movement, Energy functional, Mathematical ecology, Nonlocal advection, Partial differential equation, Stability, 35B36, 35B38, 35Q92, 92D25, 92D40

## Abstract

Deriving emergent patterns from models of biological processes is a core concern of mathematical biology. In the context of partial differential equations, these emergent patterns sometimes appear as local minimisers of a corresponding energy functional. Here we give methods for determining the qualitative structure of local minimum energy states of a broad class of multi-species nonlocal advection–diffusion models, recently proposed for modelling the spatial structure of ecosystems. We show that when each pair of species respond to one another in a symmetric fashion (i.e. via mutual avoidance or mutual attraction, with equal strength), the system admits an energy functional that decreases in time and is bounded below. This suggests that the system will eventually reach a local minimum energy steady state, rather than fluctuating in perpetuity. We leverage this energy functional to develop tools, including a novel application of computational algebraic geometry, for making conjectures about the number and qualitative structure of local minimum energy solutions. These conjectures give a guide as to where to look for numerical steady state solutions, which we verify through numerical analysis. Our technique shows that even with two species, multi-stability with up to four classes of local minimum energy states can emerge. The associated dynamics include spatial sorting via aggregation and repulsion both within and between species. The emerging spatial patterns include a mixture of territory-like segregation as well as narrow spike-type solutions. Overall, our study reveals a general picture of rich multi-stability in systems of moving and interacting species.

## Introduction

A central purpose of mathematical biology is to provide a way of linking biological processes to emergent patterns (Levin 1992; Murray 2001). In cell biology, such insights can illuminate the mechanisms behind the growth of cancerous tumours, and inform the development of interventions to slow or halt that growth (Altrock et al. 2015; Byrne 2010; Painter and Hillen 2013). In ecology, the insights on mechanisms behind animal space use can be valuable for species conservation (Bellis et al. 2004; Macdonald and Rushton 2003; Zeale et al. 2012), ensuring maintenance of biodiversity (Hirt et al. 2021; Jeltsch et al. 2013), and controlling biological invasions (Hastings et al. 2005; Lewis et al. 2016; Shigesada and Kawasaki 1997).

For partial differential equation (PDE) models of biological systems, one useful method to link process to pattern is to construct an energy functional for a system, if it exists. Then the local minima of this energy functional give possible final configurations of the system. Our focus here is to develop techniques for finding such local energy minima in a particular system of PDEs describing symmetric nonlocal multi-species interactions, with the parallel biological aim of being able to detect and describe the possible long-term patterns that may emerge from underlying processes.

The PDE system we focus on is a multi-species system of nonlocal advection diffusion equations recently introduced (Potts and Lewis 2019) and slightly generalised by Giunta et al. (2021a). This system models the spatial structure of ecosystems over timescales where births and deaths are negligible and has the following functional form

1 $$\begin{aligned} \frac{\partial u_i}{\partial t}=D_i \Delta u_i+\nabla \cdot \left( u_i \sum _{j=1}^{N}\gamma _{i j} \nabla (K *u_j) \right) , \end{aligned}$$

for $i\in \{1,\ldots ,N\}$, where $D_i$ and $\gamma _{ij}$ are constants, and $u_i(x,t)$ is the density of a species of moving organisms in location *x* at time *t*. Individuals detect the presence of others over a spatial neighborhood described by spatial averaging kernel *K*, which is a symmetric, non-negative function with $\Vert K\Vert _{L^1}=1$. The magnitude of $\gamma _{ij}$ gives the rate at which species *i* advects towards (resp. away) from species *j* if $\gamma _{ij}<0$ (resp. $\gamma _{ij}>0$). Whilst the detection of individuals may be direct, e.g. through sight smell or sound, Potts and Lewis (2019) showed that the above formalism can also be used when interactions are mediated by marks in the environment or memory of past interactions. Note that, as well as modelling different species of organism, Eq. (1) can also be used to model *N* different groups within a species, or to describe more complex situations where organisms may be spatially delineated by something other than species, e.g. mixed-species territorial flocks of birds (Mokross et al. 2018). However, we use the term ‘species’ for simplicity.

Equation (1) generalises a variety of existing models. In the case $N=1$ and $\gamma _{11}<0$, Eq. (1) is an aggregation–diffusion equation (Carrillo et al. 2018, 2019) and also arises in model of animal home ranges (Briscoe et al. 2002). For $N=2$ and $\gamma _{12},\gamma _{21}>0$, Eq. (1) can be related to models of territory formation (Ellison et al. 2020; Potts and Lewis 2016b; Rodríguez and Hu 2020) and cell sorting (Burger et al. 2018) (the latter also includes $\gamma _{12},\gamma _{21}<0$). The case of arbitrary *N* with $\gamma _{ij}=1$ has also been recently studied in the context of territories (Ellefsen and Rodríguez 2021). Finally, the $N=2$ case with $\gamma _{12}$ and $\gamma _{21}$ having different signs has been studied in the context of predator–prey dynamics (Di Francesco and Fagioli 2016). So there is a wide range of possible applications arising from Eq. (1).

Whilst our approach is quite general in potential applicability, there are various specific biological questions that might be addressed by classifying minimum energy solutions. A simple example is that of animal territory formation. How much avoidance is necessary for segregated territories to form? Is the emergence of territories history dependent? Do symmetric avoidance mechanisms always lead to symmetric territories? As another example, in the case of mutualistic species, we can ask similar questions. How much attraction is necessary for aggregation? Is it history dependent? All of these questions can benefit from the insight provided by classifying minimum energy solutions to Eq. (1), as well as more complex questions regarding multi-species questions that may exhibit a mixture of attraction and avoidance mechanisms.

The model given by Eq. (1) has been shown to exhibit rich pattern formation properties, including aggregation, segregation, oscillatory patterns and non-periodic spatio-temporal solutions suggestive of strange attractors (Potts and Lewis 2019). In Potts and Lewis (2019), for the simple case where $N=2$, $\gamma _{ii}=0$, and $\gamma _{12}=\gamma _{21}$, an energy functional was constructed that is decreasing in time, bounded below, and becomes a steady state of Eq. (1) as $t\rightarrow \infty$. Furthermore, numerical experiments suggest that only stationary patterns emerge in this case (Potts and Lewis 2019). Here, our first task is to generalise this $N=2$ energy functional to arbitrary *N*, but where $\gamma _{ij}=\gamma _{ji}$ for all $i,j,\in \{1,\ldots ,N\}$. Related work by Jüngel et al. (2022) found two more energy functionals which are based on the Shannon entropy on the one hand and a Rao-like entropy on the other. However, our focus here is on the generalization of the energy function from Potts and Lewis (2019).

Once this energy functional has been constructed, our second task is to minimise it to ascertain the functional form of the local minimum energy solutions. For this, we work in the local limit, i.e. where *K* tends towards a Dirac-$\delta$ function. We give a numerical technique for showing that, if we start with a class of stable steady state solutions for different *K*, then take the local limit, we return a piecewise constant function. This technique makes use of the theory of Gröbner bases and associated methods from computational algebraic geometry. It is a generalisation of a method first used in Potts and Lewis (2016b).

In situations where the local limit is piecewise constant, local minima of the energy functional can be found by searching through the space of piecewise constant functions. We show that this can sometimes be done analytically, using some basic examples in one spatial dimension to illustrate the methods. Even in case $N=2$, this process reveals a range of situations where there are multiple local energy minima, all of which we verify via numerics away from the local limit. Overall, the methods presented here enable users to detect local minimum energy states of Eq. (1), including multiple minima, in any situation where $\gamma _{ij}=\gamma _{ji}$.

This paper is organized as follows. We begin with linear stability analysis, in Sect. 2. This sets the stage by showing that the $\gamma _{ij}=\gamma _{ji}$ case (for all *i*, *j*) leads to stationary pattern formation at small times (from perturbations of the homogeneous steady state) as long as the species have the same-sized populations. In Sect. 3, we construct an energy functional associated with Eq. (1) in the case $\gamma _{ij}=\gamma _{ji}$ (for all *i*, *j*) and analyze its properties, particularly that it decreases in time and is bounded below. Noteably, unlike the linear analysis, this does not require the species to have the same-sized populations. This section ends with a conjecture about the structure of the attractor, which is somewhat stronger than what we are able to show in this paper, but for which we have numerical evidence to suggest it might be true. In Sect. 4, we describe our technique for finding stable steady states, assuming that the local limit of stable steady states is piecewise constant, generalising a method used in Potts and Lewis (2016a). In Sect. 5, we give a method for proving that this local limit is piecewise constant, demonstrating our proof for $N=2$ and arbitrary $\gamma _{ij}$, then for $N=3$ with specific examples of $\gamma _{ij}$.

### Notation and assumptions

We use the following notation conventions throughout. Let $S \subset \mathbb {R}^n$ be a measurable set. Then we denote the measure of *S* by $|S |$, so that

2 $$\begin{aligned} |S|=\int _{S} \textbf{1}(x) \text { dx}, \end{aligned}$$

where $\textbf{1}:\mathbb {R}^n \rightarrow \mathbb {R}$ is the constant function $\textbf{1}(x)= 1$.

Let $\Omega \subset \mathbb {R}^n$ and $f:L^p(\Omega ) \rightarrow \mathbb {R}$. We use the following norms

- $$ \Vert f \Vert _{L^p(\Omega )} =( \int _{\Omega } |f|^p)^{1/p} $$, where $$ 1\le p < \infty $$,
- $$ \Vert f\Vert _{L^{\infty }(\Omega )} = \inf \{C \ge 0 : |f(x)|\le C, \text { a.e. in } \Omega \} $$.

Let $M \in \mathbb {N}$ and $g=(g_1, g_2, \ldots , g_M):(L^p(\Omega ))^M \rightarrow \mathbb {R}$. Then we define

- $$ \Vert g\Vert _{(L^p(\Omega ))^M} = \sum _{i=1}^{M}\Vert g_i \Vert _{L^p(\Omega )} $$, where $$ 1\le p < \infty $$,
- $$ \Vert g\Vert _{(L^{\infty }(\Omega ))^M} = \max _{i=1, 2, \ldots , M} \{\Vert g_i\Vert _{L^{\infty }(\Omega )}\} $$.

To ease notation, we usually write $\Vert g \Vert _{L^{p}(\Omega )}$ instead of $\Vert g\Vert _{(L^{p}(\Omega ))^M}$, if the meaning is clear from the context. We also may drop explicit dependence on $\Omega$.

We analyze Eq. (1) on the spatial domain $\Omega =[0,L_1]\times [0,L_2]\times \cdots \times [0,L_n] \subset \mathbb {R}^n$, for $n\ge 1$, with periodic boundary conditions

3 $$\begin{aligned} u_i (x_1,\ldots ,x_N,t)|_{x_j=0}= & {} u_i (x_1,\ldots ,x_N,t)|_{x_j=L_j},\nonumber \\ \partial _{x_j}u_i (x_1,\ldots ,x_N,t)|_{x_j=0}= & {} \partial _{x_j}u_i (x_1,\ldots ,x_N,t)|_{x_j=L_j}, \end{aligned}$$

for all $i=1,\ldots , N$, $j=1, \ldots , n$ and $t \ge 0$. A spatial domain with these periodic boundary conditions is a torus and we denote it by $\mathbb {T}$. For the kernel *K* we assume that $K\in L^s (\mathbb {T})$ with $s=\frac{m}{2}$ for $m\ge 2$ and $s=1$ for $m=1$. For the non-local terms in Sects. 3 and 4 (but not Sects. 2 and 5), we assume a detailed balance for all $i,j \in \{1,\ldots ,N\}$, i.e. $\gamma _{ij}=\gamma _{ji}$. Finally, in Sects. 4 and 5 we assume $n=1$.

## Linear stability analysis

Inhomogeneous solutions of PDEs can emerge when a change in a parameter causes the loss of stability of a homogeneous steady state, leading to the formation of inhomogeneous solutions (sometimes referred to as Turing patterns after Turing (1952)), which can be either stationary or periodically oscillating in time. In this section, we will analyze the linear patterns supported by Eq. (1).

In Eq. (1), the total mass of each species *i* is conserved in time, indeed on the periodic domain $\mathbb {T}$, on which conditions (3) hold, the following identities are satisfied

4 $$\begin{aligned} \frac{d}{dt} \int _{\mathbb {T}} u_i(\textbf{x},t) \text {d}\textbf{x}=0, \quad \text {for}\quad i=1, \ldots , N, \end{aligned}$$

where $\textbf{x}=(x_1, x_2, \ldots , x_N) \in \mathbb {T}$. Hence, for all $i=1, \ldots , N$,

5 $$\begin{aligned} p_i:=\int _{\mathbb {T}} u_i (\textbf{x},t) \text {d}\textbf{x}= \int _{\mathbb {T}} u_i(\textbf{x},0) d \textbf{x},\quad \text {for all}\quad t \ge 0, \end{aligned}$$

where the constant $p_i$ is the population size of species *i*. Therefore, Eq. (1) has an homogeneous steady state

6 $$\begin{aligned} \varvec{\bar{u}}=(\bar{u}_1, \bar{u}_2, \ldots , \bar{u}_N), \quad \text {where}\quad \bar{u}_i=\frac{p_i}{|{\mathbb {T}|}},\quad \text {for}\quad i=1, \ldots , N , \end{aligned}$$

unique for each value of $p_i$ (determined by the initial condition). To study the stability of $\varvec{\bar{u}}$, we introduce the vector

7 $$\begin{aligned} \textbf{w}= (u_1 - \bar{u}_1, \ldots , u_N-\bar{u}_N) = \textbf{u}^{(0)} e^{\lambda t + i \varvec{\kappa } \cdot \textbf{x}}, \end{aligned}$$

where $\textbf{u}^{(0)}$ is a constant vector, $\lambda \in \mathbb {R}$ is the growth rate of the perturbation, $\textbf{x}=(x_1, \ldots , x_n) \in \mathbb {T}$ and $\varvec{\kappa }=(\kappa _1, \ldots , \kappa _n)$ is the wave vector, whose components are the wave numbers of the perturbation and must satisfy the boundary conditions (3). We thus have

8 $$\begin{aligned} \kappa _i=\frac{2 \pi q_{i}}{L_i},\quad \text {with}\quad q_i \in \mathbb {N},\quad \text {for}\quad i=1, \ldots , n. \end{aligned}$$

Substituting Eq. (7) into Eq. (1) and neglecting nonlinear terms, we obtain the following eigenvalue problem

9 $$\begin{aligned} \lambda (\varvec{\kappa }) \textbf{w} = |\varvec{\kappa }|^2 \mathcal {L}(\varvec{\kappa }) \textbf{w} \end{aligned}$$

where

10 $$\begin{aligned} \mathcal {L}(\varvec{\kappa })= \begin{bmatrix} -D_1 -\gamma _{11} \bar{u}_1 \hat{K}(\varvec{\kappa }) &{} -\gamma _{12} \bar{u}_1 \hat{K}(\varvec{\kappa }) &{} \dots &{} -\gamma _{1N} \bar{u}_1 \hat{K}(\varvec{\kappa }) \\ &{} &{} &{} \\ -\gamma _{21} \bar{u}_2 \hat{K}(\varvec{\kappa }) &{} -D_2 -\gamma _{22} \bar{u}_2 \hat{K}(\varvec{\kappa }) &{} \dots &{} -\gamma _{2N} \bar{u}_2 \hat{K}(\varvec{\kappa }) \\ \vdots &{} &{} &{} \\ -\gamma _{N1}\bar{u}_N \hat{K}(\varvec{\kappa }) &{} -\gamma _{N2} \bar{u}_N \hat{K}(\varvec{\kappa }) &{} \dots &{} -D_N -\gamma _{NN} \bar{u}_N \hat{K}(\varvec{\kappa }) \end{bmatrix},\nonumber \\ \end{aligned}$$

and where

$$\begin{aligned} \hat{K}(\mathbf {\varvec{\kappa }})= \int _{\mathbb {T}} K(\textbf{x}) e^{-i \varvec{\kappa } \cdot \textbf{x}} \text {d}\textbf{x}. \end{aligned}$$

For each $\varvec{\kappa }$, the eigenvalue with greatest real part (called the dominant eigenvalue) determines whether or not non-constant perturbations of the constant steady state at wavenumber $\varvec{\kappa }$ will grow or shrink at short times. If the dominant eigenvalue has positive real part and non-zero imaginary part, then these perturbations oscillate in time as they emerge. If the dominant eigenvalue is real, such oscillations will not occur at short times.

Now, if $\bar{u}_i= \bar{u}_j$ and $\gamma _{i j}=\gamma _{ji}$ for all $i,j=1,2, \ldots , N$ then $\mathcal {L}$ is symmetric, so all its eigenvalues are real (Artin 2011). Therefore non-constant perturbations of the constant steady state will not oscillate at short times. In practice, situations where the dominant eigenvalue is real and positive are often accompanied by non-constant stable steady states. Although this does not follow by necessity (Giunta et al. 2021b), this observation nonetheless suggests that this case provides a good starting point in searching for non-constant stationary patterns.

In the following sections, we will study the $\gamma _{ij}=\gamma _{ji}$ case through an energy functional analysis, showing how this can give us insights into the structure of non-constant stable steady states. It turns out that for this analysis, we do not need the additional assumption $\bar{u}_i= \bar{u}_j$.

We conclude this section by analysing the $N=2$ case in detail, to provide some results required in later sections. In this case, the characteristic polynomial of the matrix $\mathcal {L}$ is

11 $$\begin{aligned} P(\lambda )&= \lambda ^2+((\gamma _{1 1}\bar{u}_1+\gamma _{22}\bar{u}_2)\hat{K}(\varvec{\kappa })+(D_1 + D_2))\lambda \nonumber \\&\quad +(\gamma _{1 1} \gamma _{2 2}-\gamma _{1 2} \gamma _{21})\bar{u}_1 \bar{u}_2\hat{K}(\varvec{\kappa })^2 \end{aligned}$$

12 $$\begin{aligned}&\quad +(D_1 \gamma _{2 2}\bar{u}_2+D_2 \gamma _{1 1}\bar{u}_1)\hat{K}(\varvec{\kappa })+D_1 D_2, \end{aligned}$$

whose roots are

13 $$\begin{aligned} \lambda ^{\pm }(\varvec{\kappa })&=\frac{1}{2}\left[ -(\gamma _{11}\bar{u}_1+\gamma _{22}\bar{u}_2)\hat{K}(\varvec{\kappa })-(D_1+D_2)\right. \nonumber \\&\quad \left. \pm \left( ((\gamma _{11}\bar{u}_1-\gamma _{22}\bar{u}_2)^2+4 \gamma _{12}\gamma _{21}\bar{u}_1 \bar{u}_2)\hat{K}(\varvec{\kappa })^2\right. \right. \nonumber \\&\quad \left. \left. +2(D_1-D_2)(\gamma _{11}\bar{u}_1-\gamma _{22}\bar{u}_2)\hat{K}(\varvec{\kappa })+(D_1-D_2)^2\right) ^{1/2}\right] , \end{aligned}$$

giving the eigenvalues of $\mathcal {L}$. The condition $\gamma _{12}=\gamma _{21}$ ensures that the argument of the square root is always positive and therefore the eigenvalues $\lambda ^{\pm }$ are real. As a concrete example, if $p_1=p_2=1$, $L_1=\cdots =L_N=1$, $D_1=D_2$, $\gamma _{1 2}=\gamma _{2 1}$ and $\gamma _{1 1}=\gamma _{2 2}$ then the system admits a linear instability if there exists at least one $\varvec{\kappa } >0$ such that

14 $$\begin{aligned} -\gamma _{11}\hat{K}(\varvec{\kappa }) + |\gamma _{12}\hat{K}(\varvec{\kappa })|>D_1. \end{aligned}$$

## Energy functional

In this section, we will define an energy functional associated to Eq. (1) with $\gamma _{12}=\gamma _{21}$, and show that it is continuous, bounded below, decreases in time, and that its stationary points coincide with those of Eq. (1). This gives evidence to suggest that Eq. (1) with $\gamma _{12}=\gamma _{21}$ will tend towards a steady state, which will be inhomogeneous in space if the constant steady state $\varvec{\bar{u}}$ is linearly unstable.

During this section, we will assume a positivity result, namely that $u_i(x,0)>0$ implies $u_i(x,t)>0$, for all $i=1, \ldots , N$, for all $t>0$. This result has been already proved in one spatial dimension (Giunta et al. 2021a). This proof relies on a Sobolev embedding theorem only valid in one dimension, so other tools will be needed to give a proof in arbitrary dimensions. Indeed, at the time of writing, this positivity result has not yet been established in arbitrary dimensions.

First, we re-write Eq. (1) as follows

15 $$\begin{aligned} \frac{\partial u_i}{\partial t}=\nabla \cdot \left[ u_i \nabla \left( D_i \text {ln}(u_i)+\sum _{j=1}^{N}\gamma _{i j} K *u_j \right) \right] , \, i=1,\ldots , N. \end{aligned}$$

Then we define the following energy functional

16 $$\begin{aligned} E[u_1, \ldots , u_N]=\int _{\mathbb {T}} \sum _{i=1}^{N} u_i \left( D_i \text {ln}(u_i)+\frac{1}{2}\sum _{j=1}^{N}\gamma _{i j} K *u_j\right) dx, \end{aligned}$$

where $$ x=(x_1, x_2, \ldots , x_n) $$.

The first term $\sum D_i u_i \ln u_i$ is the entropy of each of the populations on their own and the second term $\sum \gamma _{ij} (K*u_j) u_i$ denotes the interaction energy between the populations (Carrillo et al. 2020). The factor $\frac{1}{2}$ before the sum is required so that we can leverage the $\gamma _{ij}=\gamma _{ji}$ symmetry later on.

### Proposition 1

The energy functional *E*, defined in Eq. (16), is a continuous function of the variables $u_1, u_2, \ldots , u_N$.

### Proof

First we show that the following functions are continuous as long as $u_i$ is positive across space and time

17 $$\begin{aligned} u_i&\longmapsto u_i \ln (u_i), \end{aligned}$$

18 $$\begin{aligned} (u_i ,u_j)&\longmapsto u_iK*u_j. \end{aligned}$$

Equation (17) is continuous since it is the product of continuous functions. For Eq. (18), we first observe that if $K \in L^1$ and $u \in L^p$, with $1\le p\le \infty$, then

19 $$\begin{aligned} \Vert K*u \Vert _{L^p} \le \Vert K\Vert _{L^1} \Vert u\Vert _{L^p}, \end{aligned}$$

by Young’s convolution inequality. Moreover, since $K *u - K*v= K*(u-v)$, we have

20 $$\begin{aligned} \Vert K*u -K *v\Vert _{L^p} = \Vert K *(u-v) \Vert _{L^p} \le \Vert K\Vert _{L^1} \Vert u-v \Vert _{L^p}=\Vert u-v \Vert _{L^p}, \end{aligned}$$

where the last equality uses $\Vert K \Vert _{L^1}=1$. Equation (20) shows that $u\mapsto K*u$ is a Lipschitz function and thus a continuous function. Therefore Eq. (18) is continuous because it is the product of continuous functions. This shows that the integrand in Eq. (16) is continuous.

Now let $1\le p\le \infty$ and $g:L^p(\Omega )\rightarrow L^p(\Omega )$ be a continuous function. Define a function $G: L^p(\Omega )\rightarrow \mathbb {R}$ by

21 $$\begin{aligned} G(u)=\int _\Omega g(u) dx. \end{aligned}$$

It remains to show that *G* is continuous. To this end, let $\epsilon >0$ and $u \in L^p(\Omega )$. Then since *g* is continuous, there exists $\delta _{\epsilon }>0$ such that for any $v \in L^p(\Omega )$ with $\Vert v-u\Vert _{{L^p}}<\delta _{\epsilon }$, we have $\Vert g(v)-g(u)\Vert _{L^{p}}<\epsilon$. Since $|G(u)-G(v)|\le \Vert g(u)-g(v)\Vert _{L^{p}}$ for all $u, v \in L^{p}(\Omega )$, we have $|G(v)-G(u)|\le \Vert g(v)-g(u)\Vert _{L^{p}}<\epsilon$. $\square$

### Remark 1

Note that whilst we have used $\Vert K \Vert _{L^1}=1$, the previous proposition also holds for any $K \in L^1$.

### Proposition 2

Suppose $\gamma _{ij}=\gamma _{ji}$, for all $i,j=1, \ldots , N$. For any positive (for each component) initial data $(u_{1,0}, \ldots , u_{N,0})$, the energy functional $E[u_1(x,t), u_2(x,t), \ldots , u_N(x,t)]$ is non-increasing over time, where $(u_1, u_2, \ldots , u_N)$ is the trajectory of Eq. (1) starting from $(u_{1,0}, \ldots , u_{N,0})$. Moreover, if *E* is constant then we are at a steady state of Eq. (1).

### Proof

Examining the time-derivative of the energy functional in Eq. (16) gives

22 $$\begin{aligned} \frac{dE}{dt}={} & {} \int _{\mathbb {T}} \sum _{i=1}^{N} \left[ \frac{\partial u_i}{\partial t} \left( D_i \text {ln}(u_i)+\frac{1}{2}\sum _{j=1}^{N}\gamma _{i j} K *u_j\right) \right. \nonumber \\ {}{} & {} \left. \qquad +u_i\left( \frac{D_i}{u_i}\frac{\partial u_i}{\partial t}+\frac{1}{2} \sum _{j=1}^{N}\gamma _{i j} K *\frac{\partial u_j}{\partial t}\right) \right] dx\nonumber \\ ={} & {} \int _{\mathbb {T}} \sum _{i=1}^{N} \left[ \frac{\partial u_i}{\partial t} \left( D_i \text {ln}(u_i)+\frac{1}{2}\sum _{j=1}^{N}\gamma _{i j} K *u_j +D_i\right) +\frac{1}{2} \sum _{j=1}^{N}\gamma _{i j} \frac{\partial u_j}{\partial t} K *u_i \right] dx \nonumber \\ ={} & {} \int _{\mathbb {T}}\left[ \sum _{i=1}^{N} \frac{\partial u_i}{\partial t} \left( D_i \text {ln}(u_i)+\frac{1}{2}\sum _{j=1}^{N}\gamma _{i j} K *u_j +D_i\right) +\frac{1}{2} \sum _{i,j=1}^{N}\gamma _{ji} \frac{\partial u_i}{\partial t} K *u_j \right] dx\nonumber \\ ={} & {} \int _{\mathbb {T}}\left[ \sum _{i=1}^{N} \frac{\partial u_i}{\partial t} \left( D_i \text {ln}(u_i)+\frac{1}{2}\sum _{j=1}^{N}\gamma _{i j} K *u_j +D_i\right) +\frac{1}{2} \sum _{i,j=1}^{N}\gamma _{ij} \frac{\partial u_i}{\partial t} K *u_j \right] dx\nonumber \\ ={} & {} \int _{\mathbb {T}}\left[ \sum _{i=1}^{N} \frac{\partial u_i}{\partial t} \left( D_i \text {ln}(u_i)+\sum _{j=1}^{N}\gamma _{i j} K *u_j +D_i\right) \right] dx\nonumber \\ ={} & {} \int _{\mathbb {T}} \sum _{i=1}^{N} \nabla \cdot \left[ u_i \nabla \left( D_i \text {ln}(u_i)+\sum _{j=1}^{N}\gamma _{i j} K *u_j\right) \right] \nonumber \\ {}{} & {} \qquad \left[ D_i\text {ln}(u_i) +\sum _{j=1}^{N}\gamma _{ij} K *u_j +D_i \right] dx. \end{aligned}$$

Here, the second equality uses that $\int _{\mathbb {T}} g (K*h) dx=\int _{\mathbb {T}} h (K*g)dx$ as long as $K(x)=K(-x)$ for $x\in \mathbb {R}^{n}$. The fourth equality uses $\gamma _{ij}=\gamma _{ji}$ and the sixth uses Eq. (15).

Before continuing the computations in Eq. (22), we simplify notation by setting

23 $$\begin{aligned} f_i= D_i\text {ln}(u_i) +\sum _{j=1}^{N}\gamma _{ij} K *u_j +D_i. \end{aligned}$$

Observing that

24 $$\begin{aligned} \nabla \cdot (u_i \nabla f_i)= \sum _{h=1}^{n} \partial _{x_h} (u_i \partial _{x_h}f_i), \end{aligned}$$

we continue the previous computation to give

25 $$\begin{aligned} \frac{dE}{dt}= & {} \int _{\mathbb {T}} \sum _{i=1}^{N} \sum _{h=1}^{n} \partial _{x_h} \left( u_i \partial _{x_h} f_i \right) f_i dx \nonumber \\= & {} \int _{\mathbb {T}} \sum _{i=1}^{N} \sum _{h=1}^{n} \left( \partial _{x_h} (u_i f_i \partial _{x_h} f_i) - u_i (\partial _{x_h} f_i)^2 \right) dx \nonumber \\= & {} -\int _{\mathbb {T}} \sum _{i=1}^{N} \sum _{h=1}^{n} u_i (\partial _{x_h} f_i)^2 dx \nonumber \\= & {} -\int _{\mathbb {T}} \sum _{i=1}^{N} u_i |\nabla f_i |^2 dx \nonumber \\= & {} -\int _{\mathbb {T}} \sum _{i=1}^{N} u_i \left|\nabla \left( D_i \text {ln}(u_i)+\sum _{j=1}^{N}\gamma _{i j} K *u_j\right) \right|^2 dx \le 0. \end{aligned}$$

The final inequality uses the assumption that $u_i > 0$. The second equality uses integration by parts. The third equality follows from the following equalities

26 $$\begin{aligned}&\int _{\mathbb {T}} \sum _{i=1}^{N} \sum _{h=1}^{n} \partial _{x_h} (u_i f_i \partial _{x_h} f_i)\nonumber \\&\quad = \int _{0}^{L_1} \int _{0}^{L_2 }\cdots \int _{0}^{L_n} \sum _{i=1}^{N} \sum _{h=1}^{n} \partial _{x_h} (u_i f_i \partial _{x_h}f_i)dx_1 dx_2\ldots dx_n\nonumber \\&\quad = \int _{0}^{L_2 }dx_2\cdots \int _{0}^{L_n}dx_n \left[ \sum _{i=1}^{N} (u_i f_i \partial _{x_1}f_i)\right] _{x_1=0}^{x_1=L_1}\nonumber \\&\qquad +\int _{0}^{L_1 }dx_1\cdots \int _{0}^{L_n}dx_n \left[ \sum _{i=1}^{N} (u_i f_i \partial _{x_2}f_i)\right] _{x_2=0}^{x_2=L_2}\nonumber \\&\qquad +\cdots +\int _{0}^{L_1 }dx_1\cdots \int _{0}^{L_{n-1}}dx_{n-1} \left[ \sum _{i=1}^{N} (u_i f_i \partial _{x_n}f_i)\right] _{x_n=0}^{x_n=L_n}, \end{aligned}$$

and we observe that each term in Eq. (26) is equal to zero due to the periodic boundary conditions in Eq. (3).

Equation (25) shows that *E* is decreasing over time unless

27 $$\begin{aligned} \nabla \left( D_i \text {ln}(u_i)+\sum _{j=1}^{N}\gamma _{i j} K *u_j\right) = 0,\quad \text {for all}\quad i=1,\ldots , N, \end{aligned}$$

which is a steady state of Eq. (15), or equivalently of Eq. (1). $\square$

### Remark 2

Proposition 2 rules out the existence of non-stationary, time-periodic solutions. Indeed, as *E* is monotonic decreasing, if there exist $t,\tau >0$ such that $E[u_1(x,t), \ldots , u_N(x,t)] = E[u_1(x,t+\tau ), \ldots , u_N(x,t+\tau )]$, then $\dot{E}(t)=0$, so Eq. (27) holds and $(u_1(x,t), \ldots , u_N(x,t))$ is a stationary solution.

### Proposition 3

Let $\Vert K\Vert _{L^{\infty }} <\infty$ and let $(u_{1,0},u_{2,0}, \ldots , u_{N,0}) \in L^1(\mathbb {T})^N$ be positive initial data and $(u_1, u_2, \ldots , u_N)$ be the trajectory of Eq. (1) starting from $(u_{1,0},u_{2,0}, \ldots , u_{N,0})$. Then $E[u_1, u_2, \ldots , u_N]$ is bounded below by a constant.

### Proof

We first observe that for all $\gamma \in \mathbb {R}$, the following inequalities hold

28 $$\begin{aligned} \int _{\mathbb {T}} \gamma u_i K*u_j dx&\ge - \left| \gamma \right| \int _{\mathbb {T}} \left| u_i K*u_j\right| dx \nonumber \\&\ge - \left| \gamma \right| \Vert u_i\Vert _{1} \Vert K*u_j\Vert _{\infty } \nonumber \\&\ge - \left| \gamma \right| \Vert u_i\Vert _{L^{1}} \Vert K\Vert _{L^{\infty }} \Vert u_j\Vert _{L^{1}}. \end{aligned}$$

The first inequality uses the fact that $\gamma \ge -\left| \gamma \right|$, for all $\gamma \in \mathbb {R}$, the second uses Hölder’s inequality and the third uses Young’s convolution inequality. Moreover, since $u_i>0$, condition (5) ensures that $\Vert u_i(x,t)\Vert _{L^1}=p_i$ for all $t \ge 0$ and thus the right-hand side of Eq. (28) is finite.

Finally, by observing that $\inf _{u_i > 0}\{u_i \text {ln}(u_i)\}=-e^{-1}$ and also by using Inequality (28), we obtain the following estimates

29 $$\begin{aligned} E[u_1, u_2, \ldots , u_N]= & {} \int _{\mathbb {T}} \sum _{i=1}^{N} u_i D_i \text {ln}(u_i) dx +\frac{1}{2}\int _{\mathbb {T}}\sum _{i,j=1}^{N}\gamma _{i j}u_i K *u_j dx \nonumber \\\ge & {} - e^{-1} |\mathbb {T} |\sum _{i=1}^{N} D_i -\frac{1}{2} \Vert K\Vert _{L^{\infty }} \sum _{i,j=1}^{N}|\gamma _{ij}|\Vert u_i\Vert _{L^1} \Vert u_j\Vert _{L^1} \nonumber \\= & {} - e^{-1} |\mathbb {T} |\sum _{i=1}^{N} D_i -\frac{1}{2} \Vert K\Vert _{L^{\infty }} \sum _{i,j=1}^{N}|\gamma _{ij}|p_i p_j, \end{aligned}$$

where the last equality uses the integral condition (5). Thus *E* is bounded below. $\square$

### Proposition 4

Suppose $$ ||K||_{L^{\infty }} < \infty $$ and $$ \gamma _{ij}=\gamma _{ji} $$, for all $$ i,j=1, \ldots , N $$. For any positive initial data $$ (u_{1,0}, \ldots , u_{N,0}) \in L^1(\mathbb {T})^N $$, there exists a constant $$ l_{u_0} $$, depending on $$ u_0$$, such that

30 $$\begin{aligned} \lim _{t\rightarrow \infty } E[u_{1}(x,t), \ldots , u_{N}(x,t)] = l_{u_0}, \end{aligned}$$

where $(u_{1}(x,t), \ldots , u_{N}(x,t))$ is the trajectory of Eq. (1) starting from $(u_{1,0}, \ldots , u_{N,0})$.

### Proof

Since $$ \Vert K\Vert _{L^{\infty }} <\infty \ $$, Prop. 3 ensures that the following set

31 $$\begin{aligned} \{E[u_{1}(x,t), \ldots , u_{N}(x,t)] : t \in \mathbb {R}^+\} \end{aligned}$$

is bounded below. Due to the Completeness Axiom of the real numbers, the set in (31) has an infimum $l_{u_0}$, which is determined by the initial condition $u_0$. Moreover, by Proposition 2, *E* is a non-increasing monotonic function of time, so tends to its infimum $l_{u_0}$ as $t\rightarrow \infty$. $\square$

Proposition 4 shows that for any initial data $$ \textbf{u}_0 \in L^1(\mathbb {T})^N $$ the trajectory starting from $$ \textbf{u}_0 $$ evolves over time towards a configuration that is a local minimiser of *E*, with energy $$E=l_{u_0}$$. We also observe that if *E* reaches the minimum value $$ l_{u_0} $$ at a finite time *T*, then the trajectory becomes stationary. Indeed, if $$ E(\textbf{u}(T))=l_{u_0} $$ then $$ E(\textbf{u}(t))\equiv l_{u_0} $$ for all $$ t \ge T $$. Hence, the minimum at $$E= l_{u_0} $$ corresponds to a steady state that is Lyapunov stable (i.e. any solution that starts arbitrarily close to the steady state will remain arbitrarily close). However, it does not guarantee asymptotic stability (i.e. any solution that starts arbitrarily close to the steady state tend toward the steady state).

In the next Section, we will propose a method to determine the structure of these minimum energy states of Eq. (1).

Finally, we note that the convergence of *E* towards a finite minimum value does not guarantee that every solution converges towards a steady state when $\gamma _{ij}=\gamma _{ji}$, as opposed to fluctuating in perpetuity. Nevertheless, this is something we would like to establish. Indeed, in all our numerical investigations, both here (in Sect. 4) and in previous works (Potts and Lewis 2019; Giunta et al. 2021a), we have only every observed (numerically) stable steady state solutions emerging, and have never observed perpetually fluctuating solutions. Therefore, we conclude this section formulating the following conjecture. This is left as an open problem, but one possible means of attack might be the via the $S^1$-equivariant theory of Buttenschön and Hillen (2021), applied there to a single-species system with a similar (but not identical) non-local advection term.

### Conjecture 5

Let $\Vert K\Vert _{L^{\infty }} < \infty$ and $\gamma _{ij}=\gamma _{ji}$, for all $i,j=1, \ldots , N$. For any positive (for each component) initial datum $u_0=(u_{1,0}, \ldots , u_{N,0}) \in L^1(\mathbb {T})^N$, the corresponding solution to Eq. (1) converges towards a steady state.

**Fig. 1:**
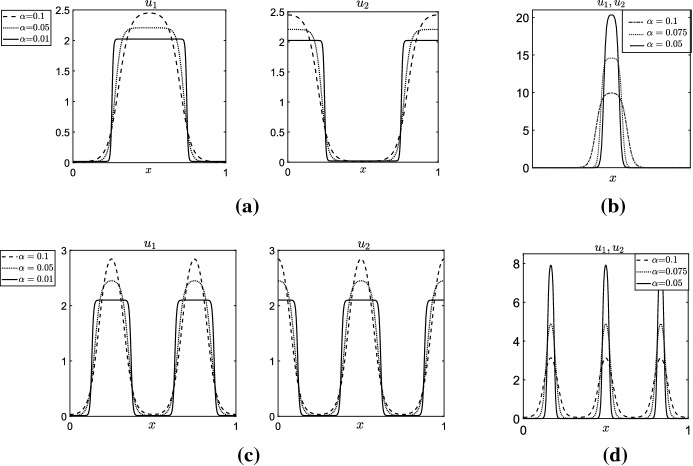
Numerical steady solutions to Eq. (1), with $N=2$, $K= K_{\alpha }(x)$ (Eq. (32)), for different values of $\alpha$. As $\alpha$ tends to zero, the solution appears to tend towards a piece-wise constant function (Panel **a** and **c**) or the limit of arbitrarily narrow, arbitrarily high piece-wise constant functions (Panel **b** and **d**). The parameter values used in the simulations are $D_1=D_2=1$, $p_1=p_2=1$, $\gamma _{11}=\gamma _{22}=0$, $\gamma _{12}=1.05$ in Panel **a** and **c**, $\gamma _{12}=-1.05$ in Panel **b** and **d**

## A method to find minimum energy states

In this section, we will propose a method to gain insight into the possible structures of minimum energy to Eq. (1). We build on methods first proposed in Potts and Lewis (2016a, Sect. 3.4) and recent existence results of Jüngel et al. (2022). We work in one spatial dimension and assume the assumptions of Sect. 1.1.

As shown in the previous section, the energy will always tend towards a local minimum, leading to a minimum energy state for the system, which is also a steady state.

When solving Eq. (1) for the top-hat kernel

32 $$\begin{aligned} K_{\alpha }(x)= {\left\{ \begin{array}{ll} \frac{1}{2 \alpha }, &{}x\in [-\alpha , \alpha ],\\ 0, &{}\text {otherwise}, \end{array}\right. } \end{aligned}$$

numerically, we find that for decreasing $\alpha$, the asymptotic steady state solutions look increasingly like piece-wise constant functions, or the limit of arbitrarily narrow, arbitrarily high piece-wise constant functions, with single or multiple peaks. These structures become more singular as $\alpha \rightarrow 0$. In Fig. 1, we see this for some simple examples. Note that as $\alpha \rightarrow 0$, the top-hat kernel in Eq. (32) becomes a Dirac delta measure, and the model (1) becomes a local cross-diffusion model. Hence we call this limit $\alpha \rightarrow 0$ as the *local limit*.

Jüngel et al. (2022) derived a solution theory for non-smooth interaction kernels *K*, which includes the case of a top-hat kernel as in Eq. (32). They consider Eq. (1) for the case where there are constants $\pi _i$ such that the matrix $(\pi _i\gamma _{ij})_{ij}$ is positive definite. For that case they showed global existence of weak solutions in Sobolev spaces. They also show a local-limit result. As $\alpha \rightarrow 0$ there exists a subsequence of solutions of Eq. (1), with *K* as in Eq. (32), that converge to a solution of the local version of Eq. (1). The norm of this convergence varies depending on the space dimension. In $n=1$ we can use any $L^p$-norm and in dimensions $n\ge 2$ we use the $L^{\frac{n}{n-1}}$-norm. These limits are piece-wise constant solutions, and spike solutions, depending on the sign of $\gamma _{ij}$. They arise as minimizers of the local version of the energy functional (Eq. (16)), which is

33 $$\begin{aligned} E_{\textrm{local}}[u_1, \ldots , u_N]=\int _{\mathbb {T}} \sum _{i=1}^{N} u_i \left( D_i \text {ln}(u_i)+\frac{1}{2}\sum _{j=1}^{N}\gamma _{i j} u_j\right) dx, \end{aligned}$$

where $$ x=(x_1, x_2, \ldots , x_n) $$.

Hence in the following we consider piece-wise constant energy minimizers, assuming that they are close to the minimizers of the non-local problem and we confirm this relation numerically. We also focus on the $n=1$ case and write $L=L_1$ for simplicity.

We now explain our method in detail. First, Eq. (25) in one dimension tells us that any minimum energy solution, $u_i (x)$, occurs when

34 $$\begin{aligned} 0= u_i \left[ \frac{\partial }{\partial x} \left( D_i \text {ln}(u_i )+\sum _{j=1}^{N}\gamma _{i j} K *u_j \right) \right] ^2, \end{aligned}$$

for each $i \in \{1,\ldots ,N\}$. Next we take the local limit of Eq. (34), which in the case $K=K_\alpha$ is the limit $\alpha \rightarrow 0$. In this limit, Eq. (34) becomes

35 $$\begin{aligned} 0 = u_i \left[ \frac{\partial }{\partial x} \left( D_i \text {ln}(u_i )+\sum _{j=1}^{N}\gamma _{i j} u_j \right) \right] ^2. \end{aligned}$$

Therefore, either $u_i (x)=0$, or, for any subinterval on which $u_i (x) \ne 0$, there exists a constant $c_i \in \mathbb {R}$ such that

36 $$\begin{aligned} c_i=D_i \text {ln}(u_i )+\sum _{j=1}^{N}\gamma _{i j} u_j,\quad \text {for}\quad i =1,\ldots ,N. \end{aligned}$$

In principle, there might exist infinitely many subintervals on which $u_i(x) \ne 0$, and $c_i$ may vary between these different subintervals. However, for each set of constants $c_1,\ldots ,c_N$, Eq. (36) will typically have a finite number of common solutions (indeed, Sect. 5 shows how to determine whether we are in this ‘typical’ situation).

Therefore, on each subinterval *I* in which $u_i(x) \ne 0$, there exists a finite set of values $u_{i1}^c, \ldots , u_{ih}^c$, with $h \in \mathbb {N}$, satisfying Eq. (36), such that

37 $$\begin{aligned} u_i (x)={\left\{ \begin{array}{ll} u_{i1}^c, &{}\text{ for }\quad x \in I_{i1}, \\ \vdots &{} \\ u_{ih}^c, &{}\text{ for }\quad x \in I_{ih}, \\ \end{array}\right. } \end{aligned}$$

where $I_{il}$, for $i=1,\ldots ,N$ and $l=1, \ldots , h$, are disjoint subsets of *I* such that $\cup _{l}I_{il}=I$ for each *i*. By considering all such subintervals *I* together, Eq. (37) defines a class of piece-wise constant functions on [0, *L*]. The aim here is to examine which of these functions is a local minimum of the energy and satisfies all model assumptions.

The general case is too complicated to deal with in one go, so we demonstrate our method on some simple examples for the case of two species, $N=2$. We start by studying the case $\gamma _{1 1} =\gamma _{22}=0$, so there is neither self-attraction nor self-repulsion. We split this analysis further into the cases of mutual avoidance ($\gamma _{12}>0$) and mutual attraction ($\gamma _{12}<0$). Then we analyze the case where $\gamma _{1 1}, \gamma _{22} \ne 0$.

### The case ${\gamma _{1 1} =\gamma _{22}=0 }$ with mutual avoidance, ${\gamma _{12}=\gamma _{21}>0}$

#### Analytic results in the local limit

Minimising the energy over the full class of functions given by Eq. (37) turns out to be too complicated. However, our numerics (see Fig. 1) suggest that the local limit (i.e. $\alpha \rightarrow 0$ in the case $K=K_\alpha$) of any solution to Eq. (1) is a function of the following form

38 $$\begin{aligned} u_i (x)={\left\{ \begin{array}{ll} u_{i}^c, &{}\text{ for }\quad x \in S_{i}, \\ 0, &{} \text{ for }\quad x \in [0,L]\backslash S_{i}, \end{array}\right. } \end{aligned}$$

where $u_{i}^c \in \mathbb {R}^{+}$ and $S_{i}$ are subsets of [0, *L*], for $i\in \{1,2\}$. Therefore we restrict our search by looking for the minimisers of the energy (Eq. (16)) in the class of piece-wise constant functions defined as in Eq. (38).

By Eq. (5), in Eq. (38) we require the following constraint

39 $$\begin{aligned} u_{i}^c|S_{i}|=p_i, \quad \text {for}\quad i=1,2, \end{aligned}$$

recalling from Eq. (2) that $|S|$ denotes the measure of a set *S*, not the cardinality, and $p_i$ denotes the total population size of species *i*. We wish to find the solutions of the form in Eq. (38), subject to Eq. (39), that are local minimisers of the energy, Eq. (16). Placing Eq. (38) into Eq. (16), and taking the spatially-local limit (i.e. $\alpha \rightarrow 0$ in the case $K=K_\alpha$), gives

40 $$\begin{aligned} E[u_1 ,u_2 ]&=\int _0^L \left( D_1 u_1 \ln (u_1) + D_2 u_2 \ln (u_2)+\gamma _{12} u_{1} u_{2} \right) dx \nonumber \\&= |S_{1}|D_1 u_{1}^c \text {ln}(u_{1}^c)+|S_{2}|D_2 u_{2}^c \text {ln}(u_{2}^c) + \gamma _{12} u_{1}^c u_{2}^c |S_{1} \cap S_{2}|\nonumber \\&=p_{1} D_1 \text {ln}(u_{1}^c)+p_{2} D_2 \text {ln}(u_{2}^c) + \gamma _{12} u_{1}^c u_{2}^c |S_{1} \cap S_{2}|, \end{aligned}$$

where the first equality uses $\gamma _{12}=\gamma _{21}$, the second equality uses Eq. (38) and the third equality uses Eq. (39).

In Eq. (40), notice that if we keep $$ |S_{1}|$$ and $$ |S_{2}|$$ fixed whilst lowering $$ |S_{1} \cap S_{2}|$$ then the energy decreases. Thus, if $$ |S_{1}|+|S_{2}|\le L $$, we can construct disjoint sets $$S_{1}$$ and $$S_{2}$$, and these will correspond to lower energy solutions than any pair of non-disjoint sets of equal measure. Furthermore, if $$ |S_{1}|+|S_{2}|> L $$, we can construct sets $$S_{1}$$ and $$S_{2}$$, such that $$|S_1\cap S_2|=|S_1|+|S_2|-L$$ and these will correspond to lower energy solutions than any other pair of sets of equal measure. Therefore henceforth, when $$ |S_{1}|+|S_{2}|\le L $$, we will assume that $$S_{1} \cap S_{2}=\emptyset $$, and when $$ |S_{1}|+|S_{2}|> L $$, we will assume that $$|S_1\cap S_2|=|S_1|+|S_2|-L$$.

To search for the local minimizers of the energy in Eq. (40), we thus define

41 $$\begin{aligned} \mathcal {E}({u}_{1}^c,{u}_{2}^c)= {\left\{ \begin{array}{ll} \sum _{i=1}^{2} p_{i} D_i \text {ln}(u_{i}^c), &{} \text {if}\quad |S_{1}|+ |S_{2}|\le L ,\\ \sum _{i=1}^{2} p_{i} D_i \text {ln}(u_{i}^c) + \gamma _{12} u_{1}^c u_{2}^c (|S_1|+|S_2|-L) , &{}\text {if}\quad |S_{1}|+ |S_{2}|> L. \end{array}\right. }\nonumber \\ \end{aligned}$$

To constrain our search, notice that Eq. (39) and $|S_i|\le L$ imply that

42 $$\begin{aligned} u_i^c=\frac{p_i}{|S_i|} \ge \frac{p_i}{|L|},\quad \text {for}\quad i=1,2. \end{aligned}$$

**Fig. 2:**
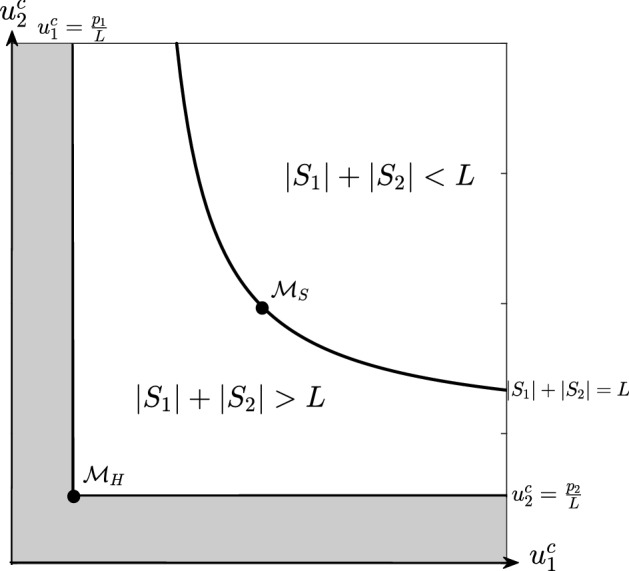
The white region represents the admissible domain, in which we look for the local minima of the function $\mathcal {E}(u_1^c,u_2^c)$ (Eq. (41)). The point $\mathcal {M}_H$, corresponding to the homogeneous steady state, is always a local minimum. Whether the point $\mathcal {M}_S$ is a local minimum depends upon the value of $\gamma _{1 2}$

The region of the $(u_1^c,u_2^c)$-plane defined by Eq. (42) is shown as white region in Fig. 2. Our strategy will be as follows. First we will look for the local minima of Eq. (41), subject to Eq. (42), in the case where $|S_1|+ |S_2|\le L$. Then we will look in the region $|S_1|+ |S_2|> L$. Combining these results will then give us a complete picture of the local minima of $\mathcal {E}(u_1^c,u_2^c)$.

Starting with $|S_1|+ |S_2|\le L$, Eq. (39) shows that this case is equivalent to the following condition

43 $$\begin{aligned} \frac{p_1}{u_1^c}+\frac{p_2}{u_2^c}=|S_1|+ |S_2|\le L. \end{aligned}$$

By analysing the partial derivatives of $$\mathcal {E}(u_1^c,u_2^c)$$ in the region of the $$(u_1^c,u_2^c)$$-plane defined by Eq. (43), we see that there are no critical points in this region. Furthermore, $$\mathcal {E}(u_1^c,u_2^c)\rightarrow \infty $$ as either $$u_1^c \rightarrow \infty $$ or $$u_2^c\rightarrow \infty $$. Therefore minima in this region must lie on the boundary, $${p_1}/{u_1^c}+{p_2}/{u_2^c}= L $$, which is shown as solid black line in Fig. 2. Analysis of the partial derivative of $$\mathcal {E}(u_1^c,u_2^c)$$ on this boundary shows that $$\mathcal {E}(u_1^c,u_2^c)$$ has a unique minimum point, given by

44 $$\begin{aligned} \mathcal {M}_S=(u_{1S}^c, u_{2S}^c):=\left( \frac{p_1 D_1+p_2 D_2}{D_1 L},\frac{p_1 D_1 +p_2D_2}{D_2 L}\right) . \end{aligned}$$

This is also a local minimum of the region defined by Eq. (43). This can be shown by performing a Taylor expansion of $\mathcal {E}(u_1^c,u_2^c)$ about the point $\mathcal {M}_S$ in the region given by $p_1/u_1^c+p_2/u_2^c \le L$. Since the slope of the tangent line to the curve $p_1/u_1^c+p_2/u_2^c = L$ at the point $\mathcal {M}_S$ is $-\frac{D_1^ 2 p_1}{D_2^2 p_2}$, we choose two arbitrarily small constants, $\epsilon$ and $\delta$, such that $D_1^ 2 p_1 \epsilon +D_2^2 p_2\delta \ge 0$ and then perform a Taylor expansion of $\mathcal {E}(u_1^c,u_2^c)$ in a neighbourhood of $\mathcal {M}_S$, which shows that

$$\begin{aligned} \mathcal {E}\left( u_{1S}^c+\epsilon ,u_{2S}^c+\delta \right)&\approx \mathcal {E}\left( u_{1S}^c,u_{2S}^c\right) + \partial _{u_1^c} \mathcal {E}\left( u_{1S}^c,u_{2S}^c\right) \epsilon + \partial _{u_2^c} \mathcal {E}\left( u_{1S}^c,u_{2S}^c\right) \delta \\&= \mathcal {E}\left( u_{1S}^c,u_{2S}^c\right) + \frac{p_1 D_1}{u_{1S}^c}\epsilon + \frac{p_2 D_2}{u_{2S}^c}\delta \\&=\mathcal {E}\left( u_{1S}^c,u_{2S}^c\right) + \frac{L}{p_1 D_1+p_2 D_2}\left( D_1^ 2 p_1 \epsilon +D_2^2 p_2\delta \right) \\&\ge \mathcal {E}\left( u_{1S}^c,u_{2S}^c\right) . \end{aligned}$$

Since $\mathcal {M}_S$ lies on the boundary curve $|S_1|+ |S_2|= L$ (Fig. 2), we have so far only established that it is a minimum of the region where $|S_1|+ |S_2|\le L$. We now need to find out whether it is a minimum for the whole admissible region (the white region in Fig. 2).

To this end, we perform a Taylor expansion of $\mathcal {E}(u_1^c,u_2^c)$ in a neighbourhood of $\mathcal {M}_S$ within the region $|S_1|+ |S_2|\ge L$, which is also the region where $p_1/u_1^c+p_2/u_2^c \ge L$, by Eq. (39). Since the slope of the tangent line to the curve $p_1/u_1^c+p_2/u_2^c = L$ at the point $\mathcal {M}_S$ is $-\frac{D_1^ 2 p_1}{D_2^2 p_2}$, we choose two arbitrary constants, $\epsilon$ and $\delta$, such that $D_1^ 2 p_1 \epsilon +D_2^2 p_2\delta \le 0$. Using Eq. (39), the function $\mathcal {E}({u}_{1}^c,{u}_{2}^c)$ in Eq. (41) becomes

45 $$\begin{aligned} \mathcal {E}(u_1^c,u_2^c)&=\sum _{i=1}^{2} p_iD_i \text {ln}(u_i^c) + \gamma _{12} u_1^c u_2^c (|S_1|+ |S_2|-L), \nonumber \\&= \sum _{i=1}^{2} p_iD_i \text {ln}(u_i^c)+ \gamma _{12} u_1^c u_2^c \left( \frac{p_1}{u_1^c} + \frac{p_2}{u_2^c} -L\right) . \end{aligned}$$

Then the Taylor expansion of $\mathcal {E}(u_1^c,u_2^c)$ in a neighbourhood of $\mathcal {M}_S$ within the region $p_1/u_1^c+p_2/u_2^c \ge L$ is

46 $$\begin{aligned} \mathcal {E}(u_{1S}^c+\epsilon ,u_{2S}^c+\delta )&\approx \mathcal {E}(u_{1S}^c,u_{2S}^c)+ \partial _{u_1^c} \mathcal {E}(u_{1S}^c,u_{2S}^c) \epsilon + \partial _{u_2^c} \mathcal {E}(u_{1S}^c,u_{2S}^c)\delta \nonumber \\&= \mathcal {E}(u_{1S}^c,u_{2S}^c) \nonumber \\&\quad + \frac{p_1 D_1}{D_2}\frac{D_1 D_2 L - \gamma _{12}(p_1 D_1 +p_2 D_2)}{p_1 D_1 +p_2 D_2} \epsilon \nonumber \\&\quad + \frac{p_2 D_2}{D_1}\frac{D_1 D_2 L - \gamma _{12}(p_1 D_1 +p_2 D_2)}{p_1 D_1 +p_2 D_2} \delta \nonumber \\&= \mathcal {E}(u_{1S}^c,u_{2S}^c) \nonumber \\&\quad + \frac{p_1 D_1^2}{D_1 D_2}\frac{D_1 D_2 L - \gamma _{12}(p_1 D_1 +p_2 D_2)}{p_1 D_1 +p_2 D_2} \epsilon \nonumber \\&\quad + \frac{p_2 D_2^2}{D_1 D_2}\frac{D_1 D_2 L - \gamma _{12}(p_1 D_1 +p_2 D_2)}{p_1 D_1 +p_2 D_2} \delta \nonumber \\&= \mathcal {E}(u_{1S}^c,u_{2S}^c) \nonumber \\&\quad + \frac{D_1 D_2 L - \gamma _{12}(p_1 D_1 +p_2 D_2)}{(D_1 D_2)(p_1 D_1 +p_2 D_2)} (D_1^ 2 p_1 \epsilon +D_2^2 p_2\delta ) \nonumber \\&\ge \mathcal {E}(u_{1S}^c,u_{2S}^c), \end{aligned}$$

if $\gamma _{12} > \frac{D_1 D_2 L}{p_1 D_1 +p_2 D_2}$, where the inequality uses $D_1^ 2 p_1 \epsilon +D_2^2 p_2\delta \le 0$.

We now examine whether there are any other minima of $\mathcal {E}(u_1^c,u_2^c)$ in the region where $|S_1|+ |S_2|> L$. By Eq. (42), the condition $|S_1|+ |S_2|> L$ is equivalent to ${p_1}/{u_1^c}+{p_2}/{u_2^c}> L$. Therefore we have the following constraints

47 $$\begin{aligned} \frac{p_1}{u_1^c}+\frac{p_2}{u_2^c}&> L, \nonumber \\ u_i^c&\ge \frac{p_i}{|L|},\quad \text {for}\quad i=1,2. \end{aligned}$$

A direct calculation using partial derivatives shows that there are no local minima of $\mathcal {E}(u_1^c,u_2^c)$ (Eq. (45)) in the interior of the region of the plane $(u_1^c, u_2^c)$ defined by Eq. (47). Therefore any local minimum must occur on the boundary. On the part of the boundary given by $u_i^c =p_i/L$, for $i=1,2$, there is a unique minimum at

48 $$\begin{aligned} \mathcal {M}_H=(u_{1H}^c, u_{2H}^c):=\left( \frac{p_1}{L},\frac{p_2}{L}\right) . \end{aligned}$$

This is also a local minimum of the region defined by Eq. (47). This can be shown by performing a Taylor expansion of $\mathcal {E}(u_1^c,u_2^c)$ about the point $\mathcal {M}_H$, to give

49 $$\begin{aligned} \nonumber \mathcal {E}(u_{1H}^c+\epsilon ,u_{2H}^c+\delta )&\approx \mathcal {E}(u_{1H}^c, u_{2H}^c)+ \partial _{u_1^c} \mathcal {E}(u_{1H}^c, u_{2H}^c) \epsilon + \partial _{u_2^c} \mathcal {E}(u_{1H}^c, u_{2H}^c)\delta \nonumber \\&= \mathcal {E}(u_{1H}^c, u_{2H}^c)+ L D_1 \epsilon + L D_2 \delta \nonumber \\&\ge \mathcal {E}(u_{1H}^c, u_{2H}^c), \end{aligned}$$

where the inequality uses $\epsilon \ge 0$, $\delta \ge 0$, so that we remain in the $u_i \ge p_i/L$ region in Fig. 2.

In summary, if $0<\gamma _{12}<\frac{D_1 D_2 L}{p_1 D_1 +p_2 D_2}$ then $\mathcal {E}(u_1^c,u_2^c)$ (Eq. (41)) has a unique minimum, given by $\mathcal {M}_H$. However, if $\gamma _{1 2}> \frac{D_1 D_2 L}{p_1 D_1 +p_2 D_2}$ then $\mathcal {E}(u_1^c,u_2^c)$ has two local minima, given by $\mathcal {M}_H$ and $\mathcal {M}_S$ (see Fig. 2).

Now, we recover the local minimizer $u_i(x)$ (Eq. (38)) of the energy (Eq. (33)). To give a concrete example, we use the parameter values $p_1=p_2=D_1=D_2=L=1$. If $(u_1^c,u_2^c)=\mathcal {M}_H$ then $u_1 (x)=u_2 (x)=1$, the homogeneous steady state, which we denote by $\mathcal {S}_H$. If $(u_1^c,u_2^c)=\mathcal {M}_S$ then

50 $$\begin{aligned} u_i (x)={\left\{ \begin{array}{ll} 2, &{}\text{ for }\quad x \in S_i \\ 0, &{} \text{ for }\quad x \in [0,1] \backslash S_i, \end{array}\right. } \end{aligned}$$

with $|S_i|=1/2$, for $i=1,2$, and $|S_1 \cap S_2|=0$. This is a class of solutions we denote by $\mathcal {S}_S^{2,2}$, where the subscript *S* stands for segregation and the superscript 2, 2 denotes the finite positive value that functions $u_1(x)$ and $u_2(x)$ take, respectively. To avoid any confusion, we want to stress that the points $\mathcal {M}_H$ (Eq. (48)) and $\mathcal {M}_S$ (Eq. (44)) are local minima of $\mathcal {E}(u_1^c,u_2^c)$ (Eq. (41)), while the functions $\mathcal {S}_H$ and $\mathcal {S}_S^{2,2}$ are minimizers of the energy $E[u_1,u_2]$ (Eq. (40)).

**Fig. 3:**
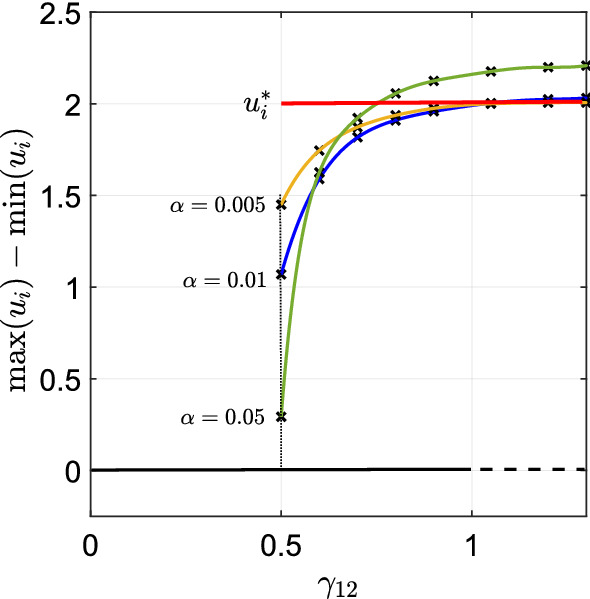
Numerically computed bifurcation diagram of Eq. (1), with $$ K=K_{\alpha }$$ (Eq. (32)), for different values of $$ \alpha $$. The other parameter values are $$p_1=p_2=D_1=D_2= L=1 $$. The red solid line shows the minimum energy branch computed analytically using the techniques in Sect. 4.1.1, pertaining to the limit $$\alpha \rightarrow 0$$. The numerical simulations show that the system admits bistability for $$ 0.5< \gamma _{12} < 1 $$, in agreement with our analytic predictions (colour figure online)

In our example, if $0<\gamma _{12}<1/2$, $E(u_1^c,u_2^c)$ (Eq. (33)) has a unique minimum, given by $\mathcal {S}_H$. If $\gamma _{1 2}>1/2$ the energy has two local minima, given by $\mathcal {S}_H$ and $\mathcal {S}_S^{2,2}$. However, recall that $\mathcal {S}_H$ and $\mathcal {S}_S^{2,2}$ are derived by minimizing the energy (Eq. (33)) in a particular class of piece-wise constant functions given by Eq. (38). Therefore, the steady states $\mathcal {S}_H$ and $\mathcal {S}_S^{2,2}$ may not be minima of the full function space where solutions might live. However, the linear stability analysis performed in Sect. 2, and particularly Eq. (14), suggests that in the limit as $\alpha$ tends to zero, $\mathcal {S}_H$ is stable if $\gamma _{12} < 1$. This gives rise to the diagram of analytically-predicted steady states given by the red and black lines in Fig. 3.

#### Numerical verification

The analysis of Sect. 4.1.1 suggests that for $p_1=p_2=D_1=D_2=L=1$, when $1/2< \gamma _{12} < 1$ and the averaging kernel *K* is arbitrarity small, Eq. (1) should exhibit bistability between the homogeneous solution, $\mathcal {S}_H$, and an inhomogeneous solution arbitrarily close to $\mathcal {S}_S^{2,2}$. Here, we verify this numerically.

Figure 3 summarises our results. To produce this figure, we start with $K=K_\alpha$ and $\gamma _{12}=1.2$, so that the homogeneous steady state is unstable. The initial condition is a small perturbation of the solution given in Eq. (50) which we run to numerical steady state. We then reduce the magnitude of $\gamma _{12}$ by $\Delta \gamma _{12}=0.05$ and solve the system again using a small random perturbation of the previous simulation as initial condition. We then repeat this process of reducing $\gamma _{12}$ and re-running to steady state until the system returns to the homogeneous steady state. This process of slowly changing one parameter and re-running to steady state is a type of numerical bifurcation analysis used in many previous studies, e.g. Painter and Hillen (2011). The numerical scheme we use for solving our particular system is detailed in Giunta et al. (2021a).

We examine three different values of $\alpha$ in Fig. 3. For each of these, we observe that the inhomogeneous solution persists below $\gamma _{12}=1$ and above $\gamma _{12}=1/2$ and, as predicted by our calculations of Sect 4.1.1, the system shows bistabilty and hysteresis. Furthermore, as $\alpha$ decreases (towards the local limit), the numerical branches appear to tend towards the branch calculated in Sect. 4.1.1.

**Fig. 4:**
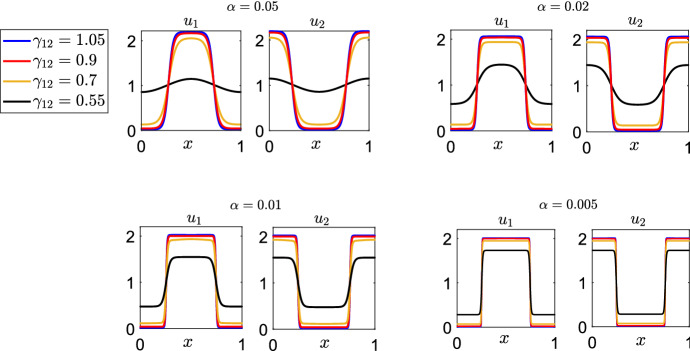
Comparison between numerically computed stationary $\mathcal {S}_S^{2,2}$ solutions to Eq. (1), with $K=K_{\alpha }$ (Eq. (32)), for different values of $\gamma _{12}>0$ and $\alpha$. The parameter values used in the simulations are $D_1=D_2=1$, $p_1=p_2=1$

Finally, in Fig. 4, we show some numerical stationary solutions for different values of $\alpha$, as $\gamma _{12}$ varies in the range [0.55, 1.05] . We observe that, as $\alpha$ decreases, the numerical solution appears to tend to a piece-wise constant function of the class given in Eq. (50) and predicted by the analysis of Sect. 4.1.1.

### The case ${\gamma _{1 1} =\gamma _{22}=0 }$ with mutual attraction, ${\gamma _{12}=\gamma _{21}<0}$

#### Analytic results in the local limit

As in Sect. 4.1.1, here we will look for the minimizers of the local version of the energy (Eq. (33)) in the class of piece-wise constant functions defined as

51 $$\begin{aligned} u_i (x)={\left\{ \begin{array}{ll} u_{i}^c, &{}\text{ for }\quad x \in S_{i}, \\ 0, &{} \text{ for }\quad x \in [0,L] \backslash S_{i}, \end{array}\right. } \end{aligned}$$

where $u_{i}^c \in \mathbb {R}^{+}$ and $S_{i}$ are subsets of [0, *L*], for $i\in \{1,2\}$.

Placing Eq. (51) into Eq. (33), and repeating the same argument of Sect. 4.1.1, we obtain

52 $$\begin{aligned} E[u_1 ,u_2 ]= \sum _{i=1}^{2} p_{i} D_i \text {ln}(u_{i}^c) + \gamma _{12} u_{1}^c u_{2}^c |S_{1} \cap S_{2}|. \end{aligned}$$

In this case, to minimize Eq. (52) we note that, since $$ \gamma _{1 2}<0 $$, $$E[u_1 ,u_2 ] $$ can be lowered by increasing $$|S_1\cap S_2|$$, whilst keeping everything else the same. Therefore if we keep $$|S_1|$$ and $$|S_2 |$$ unchanged, then $$|S_1\cap S_2|$$ is maximised when either $$S_1 \subseteq S_2$$ or $$S_2 \subseteq S_1$$, so that $$|S_1\cap S_2 |=\min _i|S_i |$$. Thus

53 $$\begin{aligned} \text{ argmin}_{u_1,u_2}E[u_1 ,u_2 ]=\text{ argmin}_{u_1,u_2}\left[ \sum _{i=1}^{2} p_iD_i \text {ln}[u_i^c]+ \gamma _{12}\min \{p_1 u_2^c,p_2u_1^c\}\right] , \end{aligned}$$

and therefore we have that $$E[u_1 ,u_2 ] \rightarrow -\infty $$ as $$\min \{p_1 u_2^c,p_2u_1^c\} \rightarrow \infty $$. As we approach this limit, $$u_1^c, u_2^c$$ become arbitrarily large, so $$u_1 $$ and $$ u_2 $$ (Eq. (51)) become arbitrarily high, arbitrarily narrow functions with overlapping support. We will denote the limit of this solution by $$ \mathcal {S}_A^{\infty } $$, in which the subscript *A* stands for aggregation and the $$\infty $$ superscript denotes that the solution becomes unbounded in the local limit. Thus $$E[u_1 ,u_2 ] $$ is minimized by $$ \mathcal {S}_A^{\infty } $$ whenever $$\gamma _{12}$$ is negative, regardless of its magnitude.

**Fig. 5:**
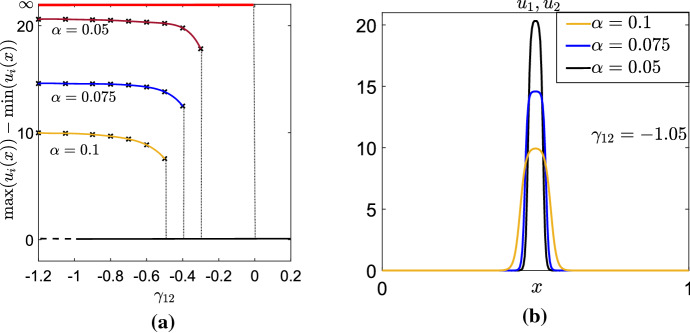
Numerical investigation of Eq. (1), with $K=K_{\alpha }$ (Equation (32)) for $\gamma _{12}<0$. The parameter values are $p_1=p_2=D_1=D_2=1$, $L=1$. Panel **a** gives a numerical bifurcation diagram showing the bistability between the homogeneous steady state (in black) and the inhomogeneous steady states $\mathcal {S}_A^{\infty }$, for different values of $\alpha$. Panel **b** shows the corresponding numerical stationary solutions whan $\gamma _{1 2}=-1.05$, for different values of $\alpha$. As $\alpha$ decreases, the solutions appear to tend towards the $\mathcal {S}_A^{\infty }$ solution

One can also show, using a very similar argument to Sect. 4.1.1 (details omitted), that the homogeneous steady state, $\mathcal {S}_H$, is the only other possible local minimiser of the energy that satisfies Eq. (42), and this is only a local minimum when $\gamma _{12}>- L(p_1 D_1 + p_2 D_2)/(p_1 p_2)$. However, linear stability analysis (Eq. (13)) suggests that, in the limit as $\alpha$ tends to zero, the homogeneous steady state is linearly stable only if $\gamma _{12} > -L \sqrt{D_1 D_2/(p_1p_2)}$. Since Young’s inequality for products implies that $L \sqrt{D_1 D_2/(p_1p_2)}< L(p_1 D_1 + p_2 D_2)/(p_1 p_2)$, any time $\mathcal {S}_H$ is linearly stable it is also a local energy minimiser within the set of functions given by Eq. (51). The red and black lines in Fig. 5a are the conclusion from combining all the results from Sect. 4.2.1, both energy functional and linear stability analysis, in the case where $p_1=p_2=D_1=D_2=L=1$.

#### Numerical verification

The analysis of Sect. 4.2.1 suggests that when $\gamma _{12} > -L \sqrt{D_1 D_2/(p_1p_2)}$, $\gamma _{12}<0$, and $\alpha$ is arbitrarily small, Eq. (1) should display bistability between the homogeneous solution and an inhomogeneous solution, whose structure tends towards $\mathcal {S}_A^{\infty }$ as $\alpha \rightarrow 0$. Here we verify this conjecture numerically, with results shown in Fig. 5a and b.

To construct these figures, we perform a similar analysis to Sect. 4.1.2. We simulate Eq. (1) with $K=K_{\alpha }$ (Eq. (32)) for small values of $\alpha$. We use the parameter values $p_1=p_2=D_1=D_2=L=1$, as in Sect. 4.1.1. For these values, the constant steady-state is stable to perturbations at all wavenumbers for $-1<\gamma _{12}<0$. Therefore, we begin our analysis by setting $\gamma _{12}=-1.2$, reducing the magnitude of $\gamma _{12}$ by a small amount ($\Delta \gamma _{12}=0.05$) at each iteration of the analysis, as in Sect. 4.1.2.

Our results show that patterns persist beyond $\gamma _{12}=-1$, and the extent of this persistence depends on $\alpha$ (Fig. 5a). As $\alpha$ is decreased, the numerical stationary states become higher, narrower functions with qualitatively similar shapes, as predicted by the previous analysis (Fig. 5b).

**Fig. 6:**
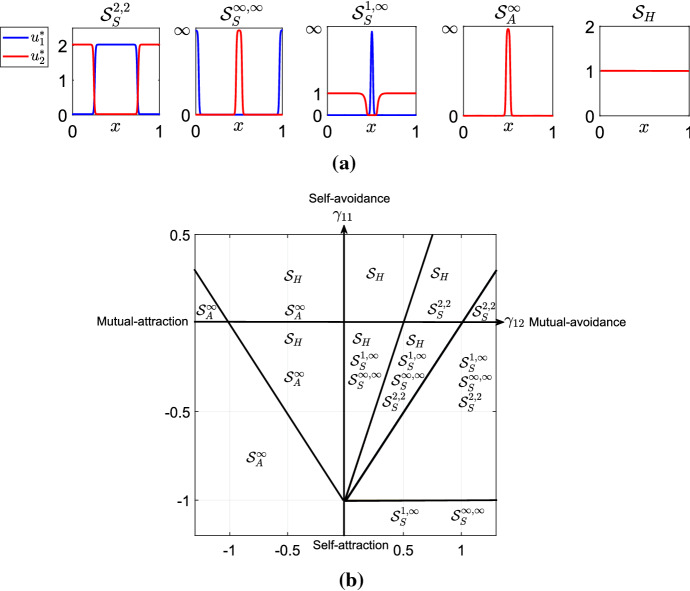
Panel **a** shows the five qualitatively-different local minimum energy states revealed by the analysis in “Appendix A”. Note that the $$ \mathcal {S}_{S}^{1,\infty } $$ solution also allows for $$u_1 $$ and $$u_2 $$ to be swapped. These plots were produced by setting $$ K=K_{\alpha } $$, $$\alpha =0.025$$ and by fixing the following parameter values: $$p_1=p_2=D_1=D_2=L=1$$. For each graph of Panel **a**, we fixed different values of the parameters $$\gamma _{11}$$ and $$\gamma _{12}$$, in particular we used: $$\gamma _{11}=0.2$$ and $$\gamma _{12}=1.05$$, for $$S_S^{2,2}$$; $$\gamma _{11}=-0.15$$ and $$\gamma _{12}=0.4$$, for $$ \mathcal {S}_S^{\infty , \infty } $$ and $$ \mathcal {S}_S^{1,\infty }$$; $$\gamma _{11}=0.2$$ and $$\gamma _{12}=-1.05$$, for $$ \mathcal {S}_A^{\infty }$$; $$\gamma _{11}=0.2$$ and $$\gamma _{12}=0.2$$, for $$ \mathcal {S}_H$$. Panel **b** shows the minimum energy solutions to Eq. (1) in different subregions of the plane $$(\gamma _{12}, \gamma _{11}) $$, for $$ N=2 $$, $$ \gamma _{2 2}=\gamma _{1 1} $$ and $$\gamma _{12} = \gamma _{21}$$, predicted by the analysis in “Appendix A”. This graph is obtained by fixing the following parameter values: $$p_1=p_2=D_1=D_2=L=1$$

### The case ${\gamma _{1 1}, \gamma _{22} \ne 0 }$

The case $\gamma _{1 1}, \gamma _{22} \ne 0$ uses similar arguments to those in Sect. 4.1. We therefore just summarise the results here, leaving details of the calculations for “Appendix A”.

In our computations, we consider the case $\gamma _{22}=\gamma _{11}$ and fix the other parameter values as $p_1=p_2=D_1=D_2=L=1$. The analysis of this case reveals five distinct classes of qualitatively-different stable solutions (Fig. 6a), each of which we have verified through numerical analysis (where throughout this section we use ‘stable’ to mean ‘Lyapunov stable’). These are (i) territory-like segregation patterns, $\mathcal {S}_S^{2,2}$, the height of which remains finite as *K* becomes arbitrarily narrow, (ii) segregation patterns where the height of both species becomes arbitrarily high as *K* becomes arbitrarily narrow, denoted by $\mathcal {S}^{\infty ,\infty }_S$, (iii) segregation patterns where the height of just one species becomes arbitrarily high as *K* becomes arbitrarily narrow but the other remains at finite height, denoted by $\mathcal {S}_S^{1,\infty }$, (iv) aggregation patterns, $\mathcal {S}_A^{\infty }$, where the height of both species becomes arbitrarily high as *K* becomes arbitrarily narrow, and (v) the spatially homogeneous solution $\mathcal {S}_H$.

**Fig. 7:**
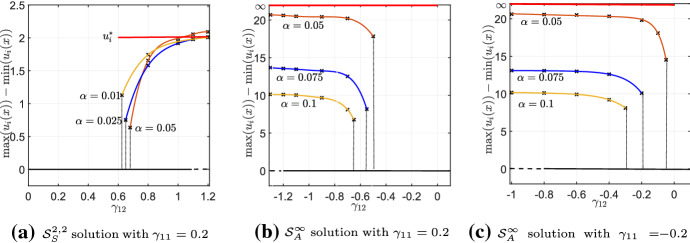
Bifurcation diagrams of Eq. (1), with $K=K_{\alpha }$ (Eq. (32)), for different values of $\gamma _{11}$, as $\alpha$ is decreased. The other parameter values are $p_1=p_2=D_1=D_2=1$, $L=1$. Panel **a** shows hysteresis between the homogeneous steady state $\mathcal {S}_H$ (in black) and the stationary solution $\mathcal {S}_S^{2,2}$ for different values of $\alpha$. As $\alpha$ decreases, the numerical branches tend towards the analytically-predicted branch (in red). Panels **b** and **c** show hysteresis between the homogeneous steady state $\mathcal {S}_H$ (in black) and the stationary solution $\mathcal {S}^{\infty }_A$ for different values of $\alpha$. As $\alpha$ decreases, the height of the numerical branches tends towards $\infty$ (colour figure online)

**Fig. 8:**
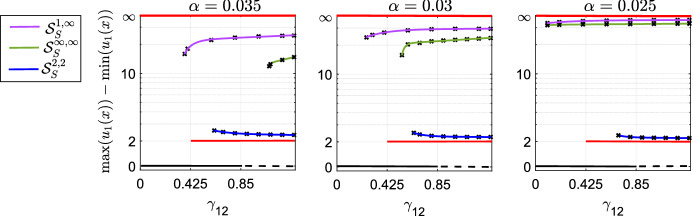
Bifurcation diagrams of Eq. (1), with $K=K_{\alpha }$ (Eq. (32)), for $\gamma _{11}=-0.15$ and different values of $\alpha$. The other parameter values are $p_1=p_2=D_1=D_2=1$, $L=1$. The graphs show the coexistence between the homogeneous steady state $\mathcal {S}_H$ (in black), computed analytically, and the stationary solutions $\mathcal {S}_S^{2,2}$ (in blue), $\mathcal {S}^{\infty }_S$ (in green) and $\mathcal {S}_{H}^{\infty }$ (in violet), computed numerically. As $\alpha$ decreases, the numerical branches tend towards the analytical branches (in red) (colour figure online)

Figure 6b shows the parameter regions in which the analysis from “Appendix A” predicts we should see these various solutions. Notice that there are regions in which we have two-, three-, and even four-fold stability. These calculations are verified numerically in Figs. 7 and 8. In particular, Figs. 7 and 8 show that, as $\alpha$ becomes smaller, so the numerical results become closer to our analytic predictions.

As shown in Fig. 6b, when species exhibit mutual attraction ($\gamma _{12}<0$), our analysis predicts two stationary states: the homogeneous distribution $S_H$ and the aggregation pattern $S_A^{\infty }$. In particular, if $\gamma _{12}<0$ and species show mutual avoidance, i.e. $\gamma _{11}>0$, there always exists a region in the parameter space in which both stationary states, $S_H$ and $S_A^{\infty }$, are stable. However, if the magnitude of self-avoidance $\gamma _{11}$ is relatively weaker than the rate of mutual-attraction $\gamma _{12}$, aggregation is more favored than the homogeneous distribution. In this case, $S_A^{\infty }$ is the only stable steady state, while the $S_H$ solution is unstable.

On the other hand, in the mutual- and self-attraction case ($\gamma _{12}<0$, $\gamma _{11}<0$), bistability between the homogeneous distribution $S_H$ and the aggregation pattern $S_A^{\infty }$ is observed as long as the magnitudes of $\gamma _{12}$ and $\gamma _{11}$ are sufficiently small. However, if the rates of mutual and self-attraction become stronger, aggregation is favoured over the homogeneous distribution. Consequently, as the magnitudes of $\gamma _{11}$ and $\gamma _{12}$ increase, the homogeneous solution $S_H$ loses stability.

The scenario becomes even richer when $\gamma _{12}>0$. In particular, if the species exhibit mutual avoidance ($\gamma _{12}>0$) and self-avoidance ($\gamma _{11}>0$), the stable steady states predicted by our analysis are the homogeneous solution $S_H$ and segregation pattern $S_S^{2,2}$. When the strength of self-repulsion ($\gamma _{11}$) is relatively stronger than the tendency to avoid individuals from the other species ($\gamma _{12}$), the homogeneous distribution is favoured over aggregation with conspecifics, so that $S_H$ is the only stable steady state. However, if the rate of mutual avoidance $\gamma _{12}$ increases, the tendency to avoid individuals from the foreign species promotes the formation of spatial distributions in which the two species are segregated into distinct sub-regions of space. Indeed, Fig. 6b shows that as $\gamma _{12}$ increases, the segregation pattern $S_S^{2,2}$ acquires stability. However, as long as the magnitude of self-avoidance is sufficiently strong, the homogeneous distribution remains stable. Indeed, we observe that there is a parameter region in which the system shows bistability between $S_H$ and $S_S^{2,2}$. Finally, if the strength of mutual avoidance $\gamma _{12}$ becomes sufficiently stronger than the propensity to avoid conspecifics, segregation becomes more favored over the homogeneous distribution. Indeed, as $\gamma _{12}$ increases, $S_H$ loses its stability.

In the mutual avoidance ($\gamma _{12}>0$) and self-attraction ($\gamma _{11}<0$) scenario, the stable states predicted by our analysis include $S_H$ (homogeneous) and $S_S^{2,2}$ (territory-like segregation) as before, but also $\mathcal {S}_S^{\infty , \infty }$ (self-aggregated species that are segregated from one another) and $\mathcal {S}_S^{1,\infty }$ (segregated species where only one population is self-aggregated). If the magnitudes of self-attraction $\gamma _{11}$ and mutual avoidance $\gamma _{12}$ are sufficiently small, the homogeneous distribution, $S_H$, is also stable. However, for small values of $\gamma _{11}$, as the rate of mutual avoidance $\gamma _{12}$ increases, we observe the same scenario discussed above: $S_S^{2,2}$ gains stability and there exists a region in the parameter space in which both $S_S^{2,2}$ and $S_H$ are stable. Finally $S_H$ loses stability as $\gamma _{12}$ increases further. We also observe that high rates of self-attraction $\gamma _{11}$ favour the formation of sub-regions with high densities of individuals. Therefore, when the magnitude of $\gamma _{11}$ is strong, $\mathcal {S}^{\infty }_S$ and $\mathcal {S}^{\infty }_H$ solutions are favored over the homogeneous distribution $S_H$ and the inhomogeneous distribution $S_S^{2,2}$, which become unstable.

Finally, we verify this multi-stability numerically for small $\alpha$, with results shown in Figs. 7 and 8. As in the $\gamma _{11}=\gamma _{22}=0$ cases, the numerics follow our analytic predictions well, giving better approximations for smaller $\alpha$.

In the following Lemma, we summarize the results shown in Fig. 6, which are derived in “Appendix A”.

#### Lemma 6

Let $\gamma _{2 2}=\gamma _{1 1}$, $\gamma _{21}=\gamma _{12}$ and $p_1=p_2=D_1=D_2=L=1$, and use ‘minimum energy’ to mean ‘local minimum energy’.

Case A: self avoidance (${\gamma _{11}>0}$) and mutual avoidance (${\gamma _{12}>0})$.

1. If $\gamma _{11} > 2\gamma _{12}-1$ then the minimum energy state is $\mathcal {S}_H$.
2. If $0<\gamma _{11} < 2\gamma _{12}-1$ then $\mathcal {S}_H$ and $\mathcal {S}_S^{2,2}$ are both minimum energy states.

Case B: Mutual attraction (${\gamma _{12}<0}$).

1. If $\gamma _{11}> -\gamma _{12}-1$ then $\mathcal {S}_H$ and $\mathcal {S}_A^{\infty }$ are minimum energy states.
2. If $\gamma _{11}< -\gamma _{12}-1$ then the minimum energy state is $\mathcal {S}_A^{\infty }$.

Case C: Self attraction (${\gamma _{11}<0}$) and mutual avoidance (${\gamma _{12}>0})$.

1. If $\gamma _{11}> 2\gamma _{12}-1$ then $\mathcal {S}_H$, $\mathcal {S}_S^{\infty ,\infty }$ and $\mathcal {S}_S^{1,\infty }$ are minimum energy states.
2. If $\gamma _{12}-1<\gamma _{11}<2 \gamma _{12}-1$ then $\mathcal {S}_H$, $\mathcal {S}_S^{\infty ,\infty }$, $\mathcal {S}_S^{1,\infty }$, and $\mathcal {S}_S^{2,2}$ are minimum energy states.
3. If $-1<\gamma _{11}< \gamma _{12}-1$ then $\mathcal {S}_S^{\infty ,\infty }$, $\mathcal {S}_S^{1,\infty }$, and $\mathcal {S}_S^{2,2}$ are minimum energy states.
4. If $\gamma _{11}< -1$ then $\mathcal {S}_S^{\infty ,\infty }$ and $\mathcal {S}_S^{1,\infty }$ are minimum energy states.

## The steady states in the local limit

In the previous section, we found piecewise constant energy minimisers of the local limit of Eq. (1). These can attain only a discrete set of values. Here, we confirm this observation by showing that, on each subinterval where the solution is differentiable, it must be constant.

For $N=2$ we prove that the image of any minimum energy solution must lie in a finite set. This proof works for any parameter values $D_i$ and $\gamma _{ij}$. We were not, however, able to prove this result in full generality for arbitrary *N*. Nonetheless, we do provide a method for constructing a proof for any particular set of parameter values, and put these ideas into practice in some example cases where $N=3$.

### The general setup

Let $K(x)=\delta (x)$, the Dirac delta function with mass concentrated at $x=0$. Then in one spatial dimension Eq. (1) becomes

54 $$\begin{aligned} \frac{\partial u_i}{\partial t}&= D_i\frac{\partial ^2 u_i}{\partial x^2} + \frac{\partial }{\partial x}\left( u_i \sum _{j=1}^N\gamma _{ij}\frac{\partial u_j}{\partial x}\right) ,\quad i=1,\ldots , N. \end{aligned}$$

Any local minimum energy solution to Eq. (54) is given by a set of functions $u_1 (x),\ldots ,u_N (x)$ that solve Eq. (27) for each $i \in \{1,\ldots ,N\}$ with $K(x)=\delta (x)$. We therefore require that, on any subinterval where $u_i(x)\ne 0$,

55 $$\begin{aligned} 0 = \frac{\textrm{d} }{\textrm{d} x}\left( D_i \text {ln}(u_i ) + \sum _{j=1}^N\gamma _{ij} u_j \right) =\frac{D_i}{u_i }\frac{\textrm{d} u_i }{\textrm{d} x} + \sum _{j=1}^N\gamma _{ij} \frac{\textrm{d} u_j }{\textrm{d} x}, \end{aligned}$$

which implies that

56 $$\begin{aligned} 0&= D_i\frac{\textrm{d} u_i }{\textrm{d} x} + u_i \sum _{j=1}^N\gamma _{ij}\frac{\textrm{d} u_j }{\textrm{d} x}. \end{aligned}$$

Equation (56) can be written in matrix form as

57 $$\begin{aligned} 0&= A_1\frac{\textrm{d} \textbf{u} }{\textrm{d} x}, \nonumber \\&\text {where } A_1:=\left( \begin{array}{cccc}D_1+\gamma _{11}u_1 &{} \gamma _{12}u_1 &{} \dots &{} \gamma _{1N}u_1 \\ \gamma _{21}u_2 &{} D_2+\gamma _{22}u_2 &{} \dots &{} \gamma _{2N}u_2 \\ \vdots &{} \vdots &{} \ddots &{} \vdots \\ \gamma _{N1}u_N &{} \gamma _{N2}u_N &{} \dots &{} D_N+\gamma _{NN}u_N \end{array}\right) , \end{aligned}$$

and $\textbf{u} =(u_1 ,\ldots ,u_N )^T$. Equation (57) holds on each subinterval where $u_i(x)\ne 0$. We wish to show that differentiable solutions are necessarily constant. Equation (57) only has a nontrivial solution if either $\det (A_1)=0$ or $\frac{\partial \textbf{u} }{\partial x}=0$. The latter means that $\textbf{u}$ is constant, so we need to investigate the condition $\det (A_1)=0$.

### The case $N=2$

To make things simple, we begin by focusing on the case $N=2$. We use the notation $A_1^{(2)}$ to mean the matrix $A_1$ (Eq. (57)) for $N=2$, so that

58 $$\begin{aligned} A_1=A_1^{(2)}:=\left( \begin{array}{cc}D_1+\gamma _{11}u_1 &{} \gamma _{12}u_1 \\ \gamma _{21}u_2 &{} D_2+\gamma _{22}u_2 \end{array}\right) . \end{aligned}$$

The condition $\det (A_1^{(2)})=0$ then implies

59 $$\begin{aligned} (D_1+\gamma _{11}u_1 )(D_2+\gamma _{22}u_2 )-\gamma _{12}\gamma _{21}u_1 u_2 =0. \end{aligned}$$

If $\textbf{u}$ is differentiable then we can differentiate Eq. (59) with respect to *x*, leading to the following

60 $$\begin{aligned}{}[\gamma _{11}(D_2+\gamma _{22}u_2 )-\gamma _{12}\gamma _{21}u_2 ]\frac{\textrm{d}u_1 }{\textrm{d}x}+[\gamma _{22}(D_1+\gamma _{11}u_1 )-\gamma _{12}\gamma _{21}u_1 ]\frac{\textrm{d}u_2 }{\textrm{d}x}=0. \end{aligned}$$

Combining Eq. (60) with the first row of the vector equation $A_1^{(2)} \frac{\textrm{d}{} \textbf{u} }{\textrm{d}x}=0$ gives

61 $$\begin{aligned} 0&= A_2^{(2)}\frac{\textrm{d} \textbf{u} }{\textrm{d} x},\quad \text{ where } \nonumber \\ A_2^{(2)}&:=\left( \begin{array}{cc}\gamma _{11}(D_2+\gamma _{22}u_2 )-\gamma _{12}\gamma _{21}u_2 &{} \; \gamma _{22}(D_1+\gamma _{11}u_1 )-\gamma _{12}\gamma _{21}u_1 \\ D_1+\gamma _{11}u_1 &{} \gamma _{12}u_1 \end{array}\right) . \end{aligned}$$

Then $\left\{ \det (A_1^{(2)})=0, \det (A_2^{(2)})=0\right\}$ is a system of two simultaneous equations in two unknowns. These have at most three solutions, as we show in “Appendix B”.

The exact form of these solutions is rather cumbersome, so we omit writing them down explicitly. However, it is instructive to give a simple example, which we do in the case $\gamma _{11}=\gamma _{22}=0$. Here, there is a single solution to $\left\{ \det (A_1^{(2)})=0, \det (A_2^{(2)})=0\right\}$ of the following form

62 $$\begin{aligned} u_1 =\frac{D_2}{\gamma _{21}},\quad u_2 =\frac{D_1}{\gamma _{12}}. \end{aligned}$$

Regardless of whether or not we impose the condition $\gamma _{11}=\gamma _{22}=0$, the solution set of $(u_1 ,u_2 )$ is a finite set. Therefore each differentiable part of a solution of Eq. (56) is constant.

### The case $N=3$

We now show how to extend the arguments of Sect. 5.2 to the $N=3$ case. The expressions become too complicated in $N=3$ to give a complete analysis, so we instead give some examples to demonstrate how one can ascertain whether or not image of $\textbf{u} (x)$ is contained in a finite set. Similar to the strategy for $N=2$, the aim is to construct a system of equations that constrain the possible solutions for $\textbf{u} (x)$. For $N=3$, this involves constructing three equations, which each take the form $\det (A_i^{(3)})=0$ for some matrix $A_i^{(3)}$ ($i \in \{1,2,3\}$), and showing that this set of simultaneous equations has a finite number of solutions. Whilst for $N=2$, we were able to calculate the number of solutions exactly by solving polynomial equations, this is not possible for $N=3$ as the polynomials are usually of order 5 or more (Stewart 2015). Instead, we use the theory of Gröbner bases to prove the solution set is finite.

#### Example 1

For this example, we let $D_i=1$, $\gamma _{ii}=0$, $\gamma _{12}=\gamma _{21}=\gamma _{23}=\gamma _{32}=2$, and $\gamma _{13}=\gamma _{31}=1$. Then

63 $$\begin{aligned} A_1=A_1^{(3)}:=\left( \begin{array}{ccc}1 &{} 2u_1 &{} u_1 \\ 2u_2 &{} 1 &{} 2u_2 \\ u_3 &{} 2u_3 &{} 1 \\ \end{array}\right) . \end{aligned}$$

Since $\det (A_1^{(3)})=0$, we have

64 $$\begin{aligned} 0=1+8 u_1 u_2 u_3 -4u_1 u_2 -4u_2 u_3 -u_1 u_3 . \end{aligned}$$

Again, assuming $\textbf{u}$ is differentiable, we can differentiate Eq. (64) with respect to *x* which leads to the following

65 $$\begin{aligned} 0&=\frac{\textrm{d}u_1 }{\textrm{d}x}(8 u_2 u_3 -4u_2 -u_3 )+\frac{\textrm{d}u_2 }{\textrm{d}x}(8 u_1 u_3 -4u_1 -4u_3)\nonumber \\&\quad +\frac{\textrm{d}u_3 }{\textrm{d}x}(8 u_1 u_2 -u_1 -4u_2 ). \end{aligned}$$

Combining Eq. (65) with the first two rows of $A_1^{(3)} \frac{\textrm{d}{} \textbf{u} }{\textrm{d}x}=0$ gives

66 $$\begin{aligned} 0&= A_2^{(3)}\frac{\textrm{d} \textbf{u} }{\textrm{d} x},\nonumber \\&\text {where } A_2^{(3)}:=\left( \begin{array}{ccc} 8 u_2 u_3 -4u_2 -u_3 &{} 8 u_1 u_3 -4u_1 -4u_3 &{}8 u_1 u_2 -u_1 -4u_2 \\ 1 &{} 2u_1 &{} u_1 \\ 2u_2 &{} 1 &{} 2u_2 \end{array}\right) . \end{aligned}$$

Once again, we have that $\det (A_2^{(3)})=0$, leading to the following polynomial equation

67 $$\begin{aligned} 0&=-u_1 -4 u_2 +20 u_1 u_2 -4 (u_1 )^2 u_2 - 32 (u_1 )^2 (u_2 )^2 + u_1 u_3 + 8 u_2 u_3 \nonumber \\&\quad -36 u_1 u_2 u_3 + 16 (u_1 )^2 u_2 u_3 +32 u_1 (u_2 )^2 u_3. \end{aligned}$$

Differentiating Eq. (67) with respect to *x* gives

68 $$\begin{aligned} 0&=\frac{\textrm{d}u_1 }{\textrm{d}x}B_1(u_1 ,u_2 ,u_3 )+\frac{\textrm{d}u_2 }{\textrm{d}x}B_2(u_1 ,u_2 ,u_3 )+\frac{\textrm{d}u_3 }{\textrm{d}x}B_3(u_1 ,u_2 ,u_3 ), \end{aligned}$$

where

69 $$\begin{aligned} B_1(u_1 ,u_2 ,u_3 )=&-1 +20 u_2 -8 u_1 u_2 -64 u_1 (u_2 )^2 + u_3 -36 u_2 u_3 \nonumber \\&+32 u_1 u_2 u_3 +32 (u_2 )^2 u_3 \end{aligned}$$

70 $$\begin{aligned} B_2(u_1 ,u_2 ,u_3 )=&-4 + 20 u_1 -4 (u_1 )^2 - 64 (u_1 )^2 u_2 + 8 u_3 - 36 u_1 u_3 \nonumber \\&+ 16 (u_1 )^2 u_3 + 64 u_1 u_2 u_3 \end{aligned}$$

71 $$\begin{aligned} B_3(u_1 ,u_2 ,u_3 )=&u_1 -36 u_1 u_2 + 16 (u_1 )^2 +32 u_1 (u_2 )^2. \end{aligned}$$

Combining Eq. (68) with the first two rows of $A_2^{(3)} \frac{\textrm{d}{} \textbf{u} }{\textrm{d}x}=0$ gives

72 $$\begin{aligned} 0&= A_3^{(3)}\frac{\textrm{d} \textbf{u} }{\textrm{d} x},\quad \text{ where } \nonumber \\ A_3^{(3)}&:=\left( \begin{array}{ccc} B_1(u_1 ,u_2 ,u_3 ) &{} B_2(u_1 ,u_2 ,u_3 ) &{} B_3(u_1 ,u_2 ,u_3 ) \\ 8 u_2 u_3 -4u_2 -u_3 &{}8 u_1 u_3 -4u_1 -4u_3 &{}8 u_1 u_2 -u_1 -4u_2 \\ 1 &{} 2u_1 &{} u_1 \end{array}\right) . \end{aligned}$$

We now have a set of three polynomials

73 $$\begin{aligned} S=\left\{ \det (A_1^{(3)}),\det (A_2^{(3)}),\det (A_3^{(3)})\right\} \end{aligned}$$

such that the image of $\textbf{u} (x)$ must lie on the common zeros of this set. In the $N=2$ case (Sect. 5.2), we had just two polynomials, both of which were cubics, thus it is possible to find formulae for the common zeros. Here, however, we have a polynomial of degree six ($\det (A_3^{(3)})$). Since there is no general solution to a sixth degree polynomial (Stewart 2015), we cannot solve the system $\det (A_1^{(3)})=0,\det (A_2^{(3)})=0,\det (A_3^{(3)})=0$ directly.

Instead, we use a classical result from algebraic geometry, which says that the number of common zeros of *S* is finite iff for each $i \in \{1,2,3\}$, the Gröbner basis of the ideal *I*(*S*) generated by *S* contains a polynomial whose leading monomial is a power of $u_i$ (Adams and Loustaunau 1994). Computation of the Gröbner basis of an ideal generated by a set of polynomials is an algorithmic procedure that is encoded into various mathematical packages, such as Mathematica (Wolfram et al. 1999) or Macauley2 (Eisenbud et al. 2013).

We use Mathematica to calculate the Gröbner basis of *I*(*S*). The result is a set of five polynomials whose leading monomials are $\beta _1 u_3^{19}$, $\beta _2 u_2 u_3^2$, $\beta _3 u_2^2 u_3$, $\beta _4 u_2^4$ and $\beta _5 u_1$, where $\beta _1,\ldots ,\beta _5$ are constants (some of which are of the order $10^{26}$ so we refrain from writing down their exact numerical values). For each *i*, there is a polynomial in the Gröbner basis whose leading monomial is a power of $u_i$. Therefore, the common zeros of *S* are finite and the image of $\textbf{u} (x)$ is contained in a finite set. Since we have assumed $\textbf{u} (x)$ is differentiable, it must also be constant.

#### Example 2

In the previous example, we were able to show that the image of $\textbf{u} (x)$ is contained in a finite set by showing it lies on the intersection of three polynomials, which is the minimum number of polynomials required in the case $N=3$. However, sometimes three polynomials is not enough. Here, we detail an example which requires the construction of five polynomials to ensure the intersection of their zeros is a finite set.

Suppose $D_i=1$, $\gamma _{ii}=0$, and $\gamma _{ij}=2$ for all $i,j \in \{1,2,3\}$ where $i \ne j$. Then

74 $$\begin{aligned} A_1=A_1^{(3)}:=\left( \begin{array}{ccc}1 &{} 2u_1 &{} 2u_1 \\ 2u_2 &{} 1 &{} 2u_2 \\ 2u_3 &{} 2u_3 &{} 1 \\ \end{array}\right) . \end{aligned}$$

Since $\det (A_1^{(3)})=0$, we have

75 $$\begin{aligned} 0=1+16 u_1 u_2 u_3 -4u_1 u_2 -4u_1 u_3 -4u_2 u_3 . \end{aligned}$$

Differentiating Eq. (75) with respect to *x* leads to the following

76 $$\begin{aligned} 0=\frac{\textrm{d}u_1 }{\textrm{d}x}(4 u_2 u_3 -u_2 -u_3 )+\frac{\textrm{d}u_2 }{\textrm{d}x}(4 u_1 u_3 -u_1 -u_3 )+\frac{\textrm{d}u_3 }{\textrm{d}x}(4 u_1 u_2 -u_1 -u_2 ) \end{aligned}$$

Combining Eq. (76) with the first two rows of $A_1^{(3)} \frac{\textrm{d}{} \textbf{u} }{\textrm{d}x}=0$ gives

77 $$\begin{aligned} 0&= A_2^{(3)}\frac{\textrm{d} \textbf{u} }{\textrm{d} x},\quad \text{ where } \nonumber \\ A_2^{(3)}&:=\left( \begin{array}{ccc} 4 u_2 u_3 -u_2 -u_3 &{}4 u_1 u_3 -u_1 -u_3 &{}4 u_1 u_2 -u_1 -u_2 \\ 1 &{} 2u_1 &{} 2u_1 \\ 2u_2 &{} 1 &{} 2u_2 \end{array}\right) . \end{aligned}$$

Once again, we have that $\det (A_2^{(3)})=0$, leading to the following polynomial equation

78 $$\begin{aligned} 0&=(4 u_2 u_3 -u_2 -u_3 )(4u_1 u_2 -2u_1 )+(4u_1 u_3 -u_1 -u_3 )(4u_1 u_2 -2u_2 )\nonumber \\&\quad +(4 u_1 u_2 -u_1 -u_2 )(1-4u_1 u_2 ). \end{aligned}$$

Differentiating Eq. (78) with respect to *x* gives

79 $$\begin{aligned} 0&=\frac{\textrm{d}u_1 }{\textrm{d}x}B_1(u_1 ,u_2 ,u_3 )+\frac{\textrm{d}u_2 }{\textrm{d}x}B_2(u_1 ,u_2 ,u_3 )+\frac{\textrm{d}u_3 }{\textrm{d}x}B_3(u_1 ,u_2 ,u_3 ), \end{aligned}$$

where

80 $$\begin{aligned} B_1(u_1 ,u_2 ,u_3 )&=(4 u_2 u_3 -u_2 -u_3 )(4 u_2 -2)+(4 u_3 -1)(4u_1 u_2 -2u_2 )\nonumber \\&\qquad +(4 u_1 u_3 -u_1 -u_3 )4u_2 +(4u_2 -1)(1-4u_1 u_2 )\nonumber \\&\qquad -(4u_1 u_2 -u_1 -u_2 )4u_2 \end{aligned}$$

81 $$\begin{aligned} B_2(u_1 ,u_2 ,u_3 )&=(4u_3 -1)(4u_1 u_2 -2u_1 )+(4u_2 u_3 -u_2 -u_3 )4u_1 \nonumber \\&\qquad + (4 u_1 u_3 -u_1 -u_3 )(4 u_1 -2)+(4u_1 -1)(1-4u_1 u_2 )\nonumber \\&\qquad -(4u_1 u_2 -u_1 -u_2 )4u_1 \end{aligned}$$

82 $$\begin{aligned} B_3(u_1 ,u_2 ,u_3 )&=(4u_2 -1)(4u_1 u_2 -2u_1 )+(4u_1 -1)(4u_1 u_2 -2u_2 ) \end{aligned}$$

Combining Eq. (79) with the first two rows of $A_2^{(3)} \frac{\textrm{d}{} \textbf{u} }{\textrm{d}x}=0$ gives

83 $$\begin{aligned} 0&= A_3^{(3)}\frac{\textrm{d} \textbf{u} }{\textrm{d} x},\quad \text{ where } \nonumber \\ A_3^{(3)}&:=\left( \begin{array}{ccc} B_1(u_1 ,u_2 ,u_3 ) &{} B_2(u_1 ,u_2 ,u_3 ) &{} B_3(u_1 ,u_2 ,u_3 ) \\ 4 u_2 u_3 -u_2 -u_3 &{}4 u_1 u_3 -u_1 -u_3 &{}4 u_1 u_2 -u_1 -u_2 \\ 1 &{} 2u_1 &{} 2u_1 \end{array}\right) . \end{aligned}$$

We now have a set of three polynomials $$S=\left\{ \det (A_1^{(3)}),\det (A_2^{(3)}),\det (A_3^{(3)})\right\} $$, such that the image of $$\textbf{u} (x)$$ must lie on the common zeros of this set. The Gröbner basis of *I*(*S*) contains eight polynomials whose leading terms are $$\beta _1 u_2 u_3^9$$, $$\beta _2 u_2 u_3^8$$, $$\beta _3 u_2 u_3^8$$, $$\beta _4 u_2 u_3^8$$, $$\beta _5 u_2 u_3^8$$, $$\beta _6 u_2 u_3^8$$, $$\beta _7 u_2 u_3^8$$, $$\beta _8 u_2 u_3^8$$ for constants $$\beta _1,\ldots ,\beta _8$$. Here, the Gröbner basis of *I*(*S*) does not contain a polynomial a with leading monomial that is a power of $$u_i $$ for any $$i=1,2,3$$, so the common zeros of *S* do not necessarily form a finite set. Therefore we need to search for further polynomials on which the solution lies, to see if we can constrain the solutions into a finite set.

To this end, we combine Eq. (76) with the first and the third row of $A_1^{(3)} \frac{\textrm{d}{} \textbf{u} }{\textrm{d}x}=0$ to give

84 $$\begin{aligned} 0&= A_{22}^{(3)}\frac{\textrm{d} \textbf{u} }{\textrm{d} x},\quad \text{ where } \nonumber \\ A_{22}^{(3)}&:=\left( \begin{array}{ccc} 4 u_2 u_3 -u_2 -u_3 &{}4 u_1 u_3 -u_1 -u_3 &{}4 u_1 u_2 -u_1 -u_2 \\ 1 &{} 2u_1 &{} 2u_1 \\ 2u_3 &{} 2u_3 &{} 1 \end{array}\right) . \end{aligned}$$

Since $\det (A_{22}^{(3)})=0$, we have

85 $$\begin{aligned} 0&=u_1 -2 u_1 u_2 + u_3 - 8 u_1 u_3 - 2 u_2 u_3 + 24 u_1 u_2 u_3\nonumber \\ {}&\quad -16 u_1^2 u_2 u_3-16 u_1^2 u_3^2-16 u_1 u_2 u_3^2. \end{aligned}$$

Differentiating Eq. (85) with respect to *x* gives

86 $$\begin{aligned} 0&=\frac{\textrm{d}u_1 }{\textrm{d}x}B_{12}(u_1 ,u_2 ,u_3 )+\frac{\textrm{d}u_2 }{\textrm{d}x}B_{22}(u_1 ,u_2 ,u_3 )+\frac{\textrm{d}u_3 }{\textrm{d}x}B_{32}(u_1 ,u_2 ,u_3 ), \end{aligned}$$

where

87 $$\begin{aligned} B_{12}(u_1 ,u_2 ,u_3 )&=1 - 2 u_2 - 8 u_3 + 24 u_2 u_3 - 32 u_1 u_2 u_3 +32 u_1 u_3^2 -16 u_2 u_3^2, \end{aligned}$$

88 $$\begin{aligned} B_{22}(u_1 ,u_2 ,u_3 )&=-2 u_2 -2 u_3 +24 u_1 u_3 -16u_1^2 u_3 -16 u_1 u_3^2, \end{aligned}$$

89 $$\begin{aligned} B_{32}(u_1 ,u_2 ,u_3 )&=1 - 2 u_2 - 8 u_1 + 24 u_1 u_2 - 32 u_1 u_2 u_3 + 32 u_1^2 u_3 -16 u_1^2 u_2. \end{aligned}$$

Combining Eq. (86) with the second and third row of $A_{22}^{(3)} \frac{\textrm{d}{} \textbf{u} }{\textrm{d}x}=0$ gives

90 $$\begin{aligned} 0&= A_{32}^{(3)}\frac{\textrm{d} \textbf{u} }{\textrm{d} x},\quad \text{ where } \nonumber \\ A_{32}^{(3)}&:=\left( \begin{array}{ccc} B_{12}(u_1 ,u_2 ,u_3 ) &{} B_{22}(u_1 ,u_2 ,u_3 ) &{} B_{32}(u_1 ,u_2 ,u_3 ) \\ 1 &{} 2u_1 &{} 2u_1 \\ 2u_3 &{} 2u_3 &{} 1 \end{array}\right) . \end{aligned}$$

We now have a set of five polynomials $$S=\left\{ \det (A_1^{(3)}),\det (A_2^{(3)}),\det (A_3^{(3)}),\right. \left. \det (A_{22}^{(3)}),\det (A_{32}^{(3)})\right\} $$, such that the image of $$\textbf{u} (x)$$ must lie on the common zeros of this set. The Gröbner basis of *I*(*S*) consists of seven polynomials whose leading monomials are $$32768 u_3^9$$, $$12 u_2 u_3^2$$, $$ 6 u_2^2 u_3$$, $$ 96 u_2^3$$, $$ -18 u_1$$, $$18 u_1 u_2$$, $$12 u_1^2$$. Since, for each $$i \in \{1,2,3\}$$, this set contains a power of $$u_i$$, the common zeros of *S* are finite, and therefore the image of $$\textbf{u} (x)$$ is contained in a finite set. Hence if $$\textbf{u} (x)$$ is differentiable, it must be constant.

## Discussion

A central aim of mathematical biology is to predict emergent features of biological systems, using dynamical systems models. Stable steady states provide an important class of emergent features, so identification of these is a key task of mathematical biology. However, for nonlinear PDEs, this is not usually an easy task (Robinson and Pierre 2003). Indeed, often this is replaced by the more tractable task of examining a system’s behaviour close to the constant steady state, which enables linear or weakly nonlinear approximations. But it is the behaviour far away from the constant solution that is interesting biologically, as that is where the patterns exist that we perceive in biological systems.

Here, we have detailed a novel method to help find local minimum energy states, which are Lyapunov stable, in a system of nonlocal advection–diffusion equations for modelling *N* species (or groups) of mobile organisms, each of which move in response to the presence of others. Our study system is closely related to (and often directly generalises) a wide variety of previous models, including those for cell aggregation (Carrillo et al. 2018) and sorting (Burger et al. 2018), animal territoriality (Potts and Lewis 2016a) and home ranges (Briscoe et al. 2002), the co-movements of predators and prey (Di Francesco and Fagioli 2016), and the spatial arrangement of human criminal gangs (Alsenafi and Barbaro 2018). Therefore our results have wide applicability across various areas of the biological sciences.

Whilst analytic determination of stable steady states in PDEs remains a difficult task in general, numerical analysis always leaves the question open of whether one has found all possible steady states or whether there are more that the researcher has simply not stumbled upon. To help guide numerical investigations, we have constructed a method, combining heuristic and analytic features, that gives clues as to where stable steady states might be found in multi-species nonlocal advection–diffusion systems. We have demonstrated in a few examples that numerical investigations agree with the predictions of our method. Whilst our method does not give an analytic solution, it should be a valuable tool for finding stable steady states in biological models that can be modelled by nonlocal advection–diffusion systems.

Our method relies on constructing an energy functional for the PDE system. We were only able to do this in the case $\gamma _{ij}=\gamma _{ji}$ for all $i,j \in \{1,\ldots ,N\}$ and assuming that the kernel *K* is identical for all species. These constraints mean that each pair of species (or populations or groups) respond to one another in a symmetric fashion, either mutually avoiding or mutually attracting with identical strengths of avoidance or attraction, respectively. This generalises a recent result of Ellefsen and Rodríguez (2021) who construct an energy functional for the case where $\gamma _{ij}=1$ for all $i,j \in \{1,\ldots ,N\}$. We conjecture that this energy functional could be used to prove that the attractor of our study system is an unstable manifold of fixed points. However, we were unable to prove this here, so encourage readers to take on this challenge.

Whilst it may be possible to construct energy functionals in some example situations where $\gamma _{ij}\ne \gamma _{ji}$ for some *i*, *j*, or where the kernel is not identical for all species (we leave this as an open question), we expect that it is not possible in general, since there are situations where the numerical analysis suggests the attractors do not consist of stable steady states, but patterns that fluctuate in perpetuity (Potts and Lewis 2019). Perhaps the simplest situation where this has been observed is for $N=2$, $\gamma _{11},\gamma _{22}<0$, and $\gamma _{12}<0<\gamma _{21}$ (Giunta et al. 2021a), whereby both populations aggregate and one ‘chases’ the other across the terrain without either ever settling to a fixed location. Furthermore, to keep our analysis as simple as possible, we only applied the techniques of Sect. 4 to some concrete examples in $n=1$ spatial dimension. Nonetheless, there is no *a priori* reason why the techniques in Sect. 4 could not be extended to higher dimensions in the future.

Whilst our method is designed for application to models of *nonlocal* advection, for which there are existence and regularity results (Giunta et al. 2021a), it works by examining the *local* limit of stable solutions. The reason for this is that these solutions are piecewise constant, so we can constrain our search for the minimum energy, enabling minimisers to be found analytically. The disadvantage is that the local limit of stable solutions is not itself the steady state solution of a well-posed system of PDEs: in the local limit, Eq. (1) becomes ill-posed. More precisely, it is unstable to arbitrarily high wavenumbers whenever the pattern formation matrix has eigenvalues with positive real part. Nonetheless, we have shown that the local limit of minimum energy solutions to the nonlocal problem is a useful object to study, even if it may not itself be the steady state solution of a system of PDEs.

It would be cleaner, however, if we were able to develop theory that did not require taking this local limit. For $N=1$, Potts and Painter (2021) developed techniques that are analogous to the ones proposed here but in discrete space. In this case, the actual stable steady states of the discrete space system become amenable to analysis via an energy functional approach similar to the one proposed here. However, generalisations of this technique to $N>1$ do not appear to be trivial from our initial investigations.

Another possible way forward is to use perturbation analysis, starting with the minimum energy solutions from the local limit, studied here, and perturbing them to give solutions to the full nonlocal system. One could then minimise the energy across this class of perturbed solutions (which would no longer be piecewise constant) to find stable steady states of the nonlocal system in Eq. (1). This is quite a nontrivial extension of the present methods, which we hope to pursue in future work. One possible avenue might be to use a kernel that allows the non-local model to be transformed into a higher-order local model (Bennett and Sherratt 2019; Ellefsen and Rodríguez 2021).

Figures 3, 5, 7 and 8 show numerical bifurcation analysis of our system in certain examples. This naturally leads to questions about the nature of these bifurcations. In particular, the discontinuity in amplitude that occurs as the constant steady state loses stability is something that is also seen with subcritical pitchfork bifurcations. In this case, the stable branches may be joined to one another by an unstable branch, or some more complicated structure. It would be valuable to investigate analytically whether this is the case. Standard tools include weakly non-linear analysis and Crandall–Rabinowitz bifurcation theory, both of which have been used successfully for nonlocal advection–diffusion equations (Buttenschön and Hillen 2021; Eftimie et al. 2009).

The system we study assumes that species advect in response to the population density of other species. However, it is agnostic as to the precise mechanisms underlying this advection. Previous studies show that Eq. (1) can be framed as a quasi-equilibrium limit of various biologically-relevant processes, such as scent marking or memory (Potts and Lewis 2016a, b, 2019). This quasi-equilibrium assumption says, in effect, that the scent marks or memory map stabilise quickly compared to the probability density of animal locations. However, it would be valuable to examine the extent to which these processes might affect the emergent patterns away from this quasi-equilibrium limit. Along similar lines, it would also be valuable to examine the extent to which our results translate to the situation where we model each individual as a separate entity, as in an individual based model (IBM), rather than using a population density function, which is a continuum approximation of an IBM. We have recently begun developing tools for translating PDE analysis to the situation of individual based models, which could be useful for such analysis (Potts et al. 2022).

In summary, we have developed novel methods for finding nontrivial steady states in a class of nonlinear, nonlocal PDEs with a range of biological applications. As well as revealing complex multi-stable structures in examples of these systems, our study opens the door to various questions regarding the bifurcation structure, the effect of nonlocality, and the structure of the attractor. We believe these will lead to yet more significant, but highly fruitful, future work.

## Appendix A: Calculations for Figure 6

Here, we give details of the calculations performed to produce the plots in Fig. 6 from Sect. 4.3. The analysis is similar to that in Sects. 4.1 and 4.2, but unlike Sects. 4.1 and 4.2 we drop the assumption that $\gamma _{11}=\gamma _{22}=0$ and we keep the assumption $\gamma _{12}=\gamma _{21}$.

We will look for the local minimizers of the following energy functional, where $K=\delta$,

A1 $$\begin{aligned} E[u_1, u_2]=\int _{\mathbb {T}} \sum _{i=1}^{2} u_i \left( D_i \text {ln}(u_i)+\frac{1}{2}\sum _{j=1}^{2}\gamma _{i j} u_j\right) dx \end{aligned}$$

in the class of piece-wise constant functions defined as

A2 $$\begin{aligned} u_i (x)={\left\{ \begin{array}{ll} u_{i}^c, &{}\text{ for }\quad x \in S_{i}, \\ 0, &{} \text{ for }\quad x \in [0,L]\backslash S_{i}, \end{array}\right. } \end{aligned}$$

where $u_{i}^c \in \mathbb {R}^{+}$ and $S_{i}$ are subsets of [0, *L*], for $i\in \{1,2\}$.

Recall that, by Eq. (5), in Eq. (A2) we require the following constraint

A3 $$\begin{aligned} u_{i}^c|S_{i}|=p_i,\quad \text {for}\quad i=1,2. \end{aligned}$$

Placing Eq. (A2) into Eq. (A1) gives

A4 $$\begin{aligned} E[u_1 ,u_2 ]&=\int _0^L \left[ \sum _{i=1}^{2}\left( D_i u_{i} \text {ln}(u_{i} )+\frac{1}{2}\gamma _{ii}u_{i} ^2\right) +\gamma _{12} u_{1} u_{2} \right] dx \nonumber \\&=\sum _{i=1}^{2} |S_{i}|\left( D_i u_{i}^c \text {ln}(u_{i}^c) +\frac{1}{2}\gamma _{ii} (u_{i}^c)^2\right) + \gamma _{12} u_{1}^c u_{2}^c |S_{1} \cap S_{2}|\nonumber \\&= \sum _{i=1}^{2} p_{i}\left( D_i \text {ln}(u_{i}^c) +\frac{1}{2}\gamma _{ii} u_{i}^c\right) + \gamma _{12} u_{1}^c u_{2}^c |S_{1} \cap S_{2}|, \end{aligned}$$

where the first equality uses $\gamma _{12}=\gamma _{21}$ and the third equality uses Eq. (A3).

Since the general analysis of this case is not straightforward, we instead set $\gamma _{1 1}=\gamma _{2 2}$ and fix the other parameter values as $p_1=p_2=D_1=D_2=L=1$. Therefore Eq. (A4) becomes

A5 $$\begin{aligned} E[u_1 ,u_2 ]= \text {ln}(u_{1}^c)+ \text {ln}(u_{2}^c) +\frac{1}{2}\gamma _{11} (u_{1}^c+u_{2}^c) + \gamma _{12} u_{1}^c u_{2}^c |S_{1} \cap S_{2}|. \end{aligned}$$

In the following, we will look for the minimizers of Eq. (A5) and examine different cases demarcated by the signs of $\gamma _{11}$ and $\gamma _{12}$.

### A.1 Self avoidance (${\gamma _{11}>0}$) and mutual avoidance (${\gamma _{12}>0}$)

Since $$ \gamma _{12}>0 $$, in Eq. (A5) if we keep $$ |S_{1}|$$ and $$ |S_{2}|$$ fixed whilst lowering $$ |S_{1} \cap S_{2}|$$ then the energy decreases. Thus, whenever $$ |S_{1}|+|S_{2}|\le L =1 $$ we can choose disjoint sets $$S_{1}$$ and $$S_{2}$$ that will correspond to lower energy solutions than any pair of non-disjoint sets of equal measure. Furthermore, if $$ |S_{1}|+|S_{2}|> 1 $$, we can construct sets $$S_{1}$$ and $$S_{2}$$, such that $$|S_1\cap S_2|=|S_1|+|S_2|-1$$ and these will correspond to lower energy solutions than any other pair of sets of equal measure. Therefore, when $$ |S_{1}|+|S_{2}|\le 1 $$, we will assume that $$S_{1} \cap S_{2}=\emptyset $$, and when $$ |S_{1}|+|S_{2}|> 1 $$, we will assume that $$|S_1\cap S_2|=|S_1|+|S_2|-1$$ (as in Sect. 4.1.1).

To search for the local minimizers of the energy in Eq. (A5), we then define

A6 $$\begin{aligned} \mathcal {E}(u_1^c,u_2^c)={\left\{ \begin{array}{ll} \text {ln}(u_{1}^c)+ \text {ln}(u_{2}^c) +\frac{1}{2}\gamma _{11} (u_{1}^c+u_{2}^c), &{} \text {if}\quad |S_1|+ |S_2|\le 1, \\ \text {ln}(u_{1}^c)+ \text {ln}(u_{2}^c) +\frac{1}{2}\gamma _{11}(u_{1}^c+u_{2}^c)&{} \\ \quad + \gamma _{12} u_{1}^c u_{2}^c |S_{1} \cap S_{2}|, &{} \text {if}\quad |S_1|+ |S_2|> 1. \end{array}\right. } \end{aligned}$$

To constrain our search, notice that Eq. (A3), $p_i=1$ and $|S_i|\le L =1$ imply that

A7 $$\begin{aligned} u_i^c\ge 1,\quad \text {for}\quad i=1,2. \end{aligned}$$

We analyse $\mathcal {E}(u_1^c,u_2^c)$ (Eq. (A6)) under the constraint in Eq. (A7), first in the region where $|S_1|+ |S_2|\le 1$ and then in the region where $|S_1|+ |S_2|> 1$. By combining these results we will have a complete picture of the local minima of $\mathcal {E}(u_1^c,u_2^c)$.

Note that by Eq. (A3), the case $|S_1|+ |S_2|\le 1$ is equivalent to

A8 $$\begin{aligned} \frac{1}{u_1^c}+\frac{1}{u_2^c}\le 1. \end{aligned}$$

By analysing the partial derivatives of $\mathcal {E}(u_1^c,u_2^c)$ in the region of the $(u_1^c,u_2^c)$-plane defined by Eq. (A8), one can check that there are no local minima in this region. Furthermore, $\mathcal {E}(u_1^c,u_2^c)\rightarrow \infty$ as either $u_1^c \rightarrow \infty$ or $u_2^c\rightarrow \infty$. Therefore any minima in this region must lie on the boundary, ${1}/{u_1^c}+{1}/{u_2^c}= 1$ (solid black line in Fig. 2). Analysis of the partial derivative of $\mathcal {E}(u_1^c,u_2^c)$ on this boundary shows that $\mathcal {E}(u_1^c,u_2^c)$ has a unique minimum point, given by

A9 $$\begin{aligned} \mathcal {M}_S=(u_{1S}^c, u_{2S}^c):=\left( 2,2\right) . \end{aligned}$$

This is also a local minimum of the region defined by Eq. (A8). This can be shown by performing a Taylor expansion of $\mathcal {E}(u_1^c,u_2^c)$ about the point $\mathcal {M}_S$. Since the slope of the line tangent to the curve ${1}/{u_1^c}+{1}/{u_2^c}= 1$ in $\mathcal {M}_S$ is $-1$, we choose two constant, $\epsilon$ and $\delta$, such that $\epsilon +\delta \ge 0$ and the Taylor expansion gives

$$\begin{aligned} \mathcal {E}(2+\epsilon ,2+\delta )&\approx \mathcal {E}(2,2)+ \partial _{u_1^c} \mathcal {E}(2,2) \epsilon + \partial _{u_2^c} \mathcal {E}(2,2)\delta \\&= \mathcal {E}(2,2)+ \frac{1}{2}(1+\gamma _{1 1})(\epsilon + \delta ) \\&\ge \mathcal {E}(2,2), \end{aligned}$$

where the inequality uses $\gamma _{1 1}>0$, $\epsilon +\delta \ge 0$.

However, since the point $\mathcal {M}_S$ lies on the boundary curve $|S_1|+ |S_2|= 1$, we do not yet know whether it is a minimum for the whole admissible region defined by Eq. (A7) (white region in Fig. 2). To this end, we examine whether $\mathcal {M}_S$ is a minimum of $\mathcal {E}(u_1^c,u_2^c)$ (Eq. (A6)) in the region where $|S_1|+ |S_2|> 1$. By Eq. (A7), the condition $|S_1|+ |S_2|> 1$ is equivalent to ${1}/{u_1^c}+{1}/{u_2^c}> 1$. Therefore we have the following constraints

A10 $$\begin{aligned} \frac{1}{u_1^c}+\frac{1}{u_2^c}&> 1, \nonumber \\ u_i^c&\ge 1,\quad \text {for}\quad i=1,2. \end{aligned}$$

Since $|S_1 \cap S_2|= |S_1|+ |S_2|- 1$, when $|S_1|+ |S_2|> 1$ the function $\mathcal {E}({u}_{1}^c,{u}_{2}^c)$ (Eq. (A6)) can be rewritten as

A11 $$\begin{aligned} \mathcal {E}(u_1^c,u_2^c)&= \text {ln}(u_{1}^c)+ \text {ln}(u_{2}^c) +\frac{1}{2}\gamma _{11} (u_{1}^c+u_{2}^c)+ \gamma _{12} u_1^c u_2^c |S_1 \cap S_2|\nonumber \\&= \text {ln}(u_{1}^c)+ \text {ln}(u_{2}^c) +\frac{1}{2}\gamma _{11} (u_{1}^c+u_{2}^c) + \gamma _{12} u_1^c u_2^c (|S_1|+ |S_2|-1), \nonumber \\&= \text {ln}(u_{1}^c)+ \text {ln}(u_{2}^c) +\frac{1}{2}\gamma _{11} (u_{1}^c+u_{2}^c)+ \gamma _{12} u_1^c u_2^c \left( \frac{1}{u_1} + \frac{1}{u_2} -1\right) , \end{aligned}$$

where the third equality uses $|S_i|=\frac{1}{u_i^c}$.

To verify whether $\mathcal {M}_S$ is also a minimum on the part of the domain given by Eq. (A10), we perform a Taylor expansion of $\mathcal {E}(u_1^c,u_2^c)$ in a neighbourhood of $\mathcal {M}_S$ within the region $1/u_1^c+1/u_2^c<1$. Since the slope of the tangent line to the curve $1/u_1^c+1/u_2^c = 1$ at the point $\mathcal {M}_S$ is $-1$, we choose two arbitrary constants, $\epsilon$ and $\delta$, such that $\epsilon + \delta \le 0$. Then Taylor expansion of $\mathcal {E}(u_1^c,u_2^c)$ is

A12 $$\begin{aligned} \mathcal {E}(2+\epsilon ,2+\delta )&\approx \mathcal {E}(2,2)+ \partial _{u_1^c} \mathcal {E}(2,2) \epsilon + \partial _{u_2^c} \mathcal {E}(2,2)\delta \nonumber \\&= \mathcal {E}(2,2)+\frac{1}{2}(1+ \gamma _{11}-2\gamma _{12})(\epsilon +\delta )\nonumber \\&\ge \mathcal {E}(2,2), \end{aligned}$$

if $\gamma _{11} < 2\gamma _{12}-1$, where the inequality uses $\epsilon + \delta \le 0$.

Next, we look for any other minima in the region defined by Eq. (A10). By analysing first partial derivatives, one can show that there are no local minima of $\mathcal {E}(u_1^c,u_2^c)$ (Eq. (A11)) in the interior of this region. Therefore any local minima must occur on the boundaries. On the part of the boundary given by $u_i^c =1$, for $i=1,2$, there is a unique minimum at

A13 $$\begin{aligned} \mathcal {M}_H=(u_{1H}^c, u_{2H}^c):=\left( 1,1\right) . \end{aligned}$$

This is also a local minimum of the region defined by Eq. (A10). This can be shown by performing a Taylor expansion of $\mathcal {E}(u_1^c,u_2^c)$ about the point $\mathcal {M}_H$, to give

$$\begin{aligned} \mathcal {E}(1+\epsilon ,1+\delta )&\approx \mathcal {E}(1,1)+ \partial _{u_1^c} \mathcal {E}(1,1) \epsilon + \partial _{u_2^c} \mathcal {E}(1,1)\delta \\&= \mathcal {E}(1,1)+ \left( 1+\frac{1}{2}\gamma _{11}\right) (\epsilon +\delta ) \\&\ge \mathcal {E}(1,1), \end{aligned}$$

where the inequality uses $\gamma _{1 1}>0$, $\epsilon \ge 0$ and $\delta \ge 0$. Here, $\epsilon$ and $\delta$ are chosen to be non-negative so that we remain in the $u_i^c \ge 1$ region (Fig. 2). Therefore, if $\gamma _{11} > 2\gamma _{12}-1$, $\mathcal {E}(u_1^c,u_2^c)$ (Eq. (A5)) has a unique minimum, given by $\mathcal {M}_H$. Whilst if $0<\gamma _{11} < 2\gamma _{12}-1$, then $\mathcal {E}(u_1^c,u_2^c)$ has two local minima, given by $\mathcal {M}_H$ and $\mathcal {M}_S$.

Finally, we write down the functions $u_i(x)$ (Eq. (A2)) which locally minimize the energy $E[u_1 ,u_2 ]$ (Eq. (A4)). If $(u_1^c,u_2^c)=\mathcal {M}_H$ then $u_1 (x)=u_2 (x)=1$, the homogeneous steady state, which we denote by $\mathcal {S}_H$. If $(u_1^c,u_2^c)=\mathcal {M}_S$ then

A14 $$\begin{aligned} u_i (x)={\left\{ \begin{array}{ll} 2, &{}\text{ for }\quad x \in S_i \\ 0, &{} \text{ for }\quad x \in [0,1] \backslash S_i , \end{array}\right. } \end{aligned}$$

with $|S_i|=1/2$, for $i=1,2$, and $|S_1 \cap S_2|=0$, denoted by $\mathcal {S}_S^{2,2}$.

In conclusion, if $\gamma _{11} > 2\gamma _{12}-1$, the energy $E(u_1^c,u_2^c)$ (Eq. (A4)) has a unique minimum, given by $\mathcal {S}_H$. However, if $0<\gamma _{11} < 2\gamma _{12}-1$ the energy has two local minima, given by $\mathcal {S}_H$ and $\mathcal {S}_S^{2,2}$. Furthermore, linear stability analysis (Eq. (14)) suggests that when $\alpha$ tends to zero, the homogeneous steady state is stable if $\gamma _{11} > \gamma _{12}-1$. This gives rise to the diagram of analytically-predicted steady states given by the red and black lines in Fig. 7a.

### A.2 Mutual attraction ($\mathbf {\gamma _{12}<0}$)

In this section, we analyze the local minimizers of the energy (Eq. (A4)) for $$\gamma _{12}<0$$, $$\gamma _{11} \in \mathbb {R}$$ and $$\gamma _{12}=\gamma _{21}$$. We observe that the energy in Eq. (A4) decreases as $$ |S_{1} \cap S_{2} |$$ increases, whilst keeping everything else constant. Therefore if we keep $$|S_1|$$ and $$|S_2 |$$ unchanged, then $$|S_1\cap S_2|$$ is maximised when either $$S_1 \subseteq S_2$$ or $$S_2 \subseteq S_1$$, so that $$|S_1\cap S_2 |=\min _i|S_i |$$. Thus by repeating the same argument presented in Sect. 4.2.1 for $$ \gamma _{12}<0 $$ and $$\gamma _{11}=0$$, we see that $$E[u_1 ,u_2 ] \rightarrow -\infty $$ as $$\min \{u_1^c,u_2^c\} \rightarrow \infty $$. As we approach this limit, $$u_1^c, u_2^c$$ become arbitrarily large, so $$u_1 $$ and $$ u_2 $$ (Eq. (A2)) become arbitrarily high, arbitrarily narrow functions with overlapping support. We will denote the limit of this solution by $$ \mathcal {S}_A^{\infty } $$.

One can also show, using a very similar argument to “Appendix A.1” (details omitted), that the homogeneous steady state, $\mathcal {S}_H$, is the only other possible local minimiser of the energy that satisfies $u_i^c \ge 1$, for $i=1,2$, and this is only a local minimum when $\gamma _{12}> -\gamma _{11}-2$. However, linear stability analysis (Eq. (13)) suggests that, in the limit as $\alpha$ tends to zero, the homogeneous steady state is linearly stable only if $\gamma _{12} > -\gamma _{11}-1$. Therefore, any time $\mathcal {S}_H$ is linearly stable, it is also a local energy minimiser within the set of functions given by Eq. (A2). These results give rise to the diagram of analytically-predicted steady states given by the red and black lines in Fig. 7b–c.

### A.3 Self attraction ($\mathbf {\gamma _{11}<0}$) and mutual avoidance ($\mathbf {\gamma _{12}>0}$)

By following the same argument of “Appendix A.1”, to search for the local minimizers of the energy in Eq. (A5), we define

A15 $$\begin{aligned} \mathcal {E}(u_1^c,u_2^c)= {\left\{ \begin{array}{ll} \text {ln}(u_{1}^c)+ \text {ln}(u_{2}^c) +\frac{1}{2}\gamma _{11} (u_{1}^c+u_{2}^c), &{} \text {if}\quad |S_1|+ |S_2|\le 1, \\ \text {ln}(u_{1}^c)+ \text {ln}(u_{2}^c) +\frac{1}{2}\gamma _{11} (u_{1}^c+u_{2}^c)&{} \\ \quad + \gamma _{12} u_{1}^c u_{2}^c |S_{1} \cap S_{2}|, &{} \text {if}\quad |S_1|+ |S_2|> 1. \end{array}\right. } \end{aligned}$$

We analyse $\mathcal {E}(u_1^c,u_2^c)$ (Eq. (A15)) under the constraint

A16 $$\begin{aligned} u_i^c\ge 1,\quad \text {for}\quad i=1,2, \end{aligned}$$

first when $|S_1|+ |S_2|\le 1$ and then when $|S_1|+ |S_2|> 1$. Recall that the condition in Eq. (A16) is obtained by Eq. (A3), using $p_i=1$ and $|S_i|\le L =1$.

When $$ |S_1|+ |S_2|\le 1 $$, $$\mathcal {E}(u_1^c,u_2^c)\rightarrow -\infty $$ as either $$u_1^c \rightarrow \infty $$ or $$u_2^c\rightarrow \infty $$. As we approach this limit, $$u_1^c, u_2^c$$ become arbitrarily large, so the functions $$u_1(x) $$ and $$ u_2(x) $$ (Eq. (A2)) become arbitrarily high, arbitrarily narrow functions with $$|S_1 \cap S_2 |= \emptyset $$. We denote the limit of this solution by $$ \mathcal {S}_S^{\infty ,\infty } $$, in which the subscript *S* stands for aggregation and the $$\infty ,\infty $$ superscript denotes that both $$u_1(x) $$ and $$ u_2(x) $$ become unbounded and separated as $$u_1^c,u_1^c \rightarrow \infty $$.

As discussed in “Appendix A.1”, $|S_1|+ |S_2|\le 1$ is equivalent to the following condition

A17 $$\begin{aligned} \frac{1}{u_1^c}+\frac{1}{u_2^c}\le 1. \end{aligned}$$

Thus, by analysing the partial derivatives of $\mathcal {E}(u_1^c,u_2^c)$ in the region of the $(u_1^c,u_2^c)$-plane defined by Eq. (A17), one can check that there are no local minima in the interior of this region. Analysis of the partial derivative of $\mathcal {E}(u_1^c,u_2^c)$ on the boundary $1/{u_1^c}+1/{u_2^c}=1$ shows that $\mathcal {E}(u_1^c,u_2^c)$ has a unique minimum point, given by

A18 $$\begin{aligned} \mathcal {M}_S=(u_{1S}^c, u_{2S}^c):=\left( 2,2\right) . \end{aligned}$$

This is also a local minimum of the region defined by Eq. (A17) when $\gamma _{11}>-1$. This can be shown by performing a Taylor expansion of $\mathcal {E}(u_1^c,u_2^c)$ about the point $\mathcal {M}_S$, to give

$$\begin{aligned} \mathcal {E}(2+\epsilon ,2+\delta )&\approx \mathcal {E}(2,2)+ \partial _{u_1^c} \mathcal {E}(2,2) \epsilon + \partial _{u_2^c} \mathcal {E}(2,2)\delta \\&= \mathcal {E}(2,2)+ \frac{1}{2}(1+\gamma _{1 1})(\epsilon + \delta )\\&\ge \mathcal {E}(2,2), \end{aligned}$$

where the inequality uses $\gamma _{1 1}>-1$, $\epsilon +\delta \ge 0$. We recall that $\epsilon +\delta \ge 0$ ensures that we remain in the $|S_1|+|S_2|\le 1$ region (Fig. 2).

Since the point $\mathcal {M}_S$ lies on the boundary curve $|S_1|+ |S_2|= 1$, we have so far only established that when $\gamma _{1 1}>-1$, $\mathcal {M}_S$ is a minimum of $\mathcal {E}(u_1^c,u_2^c)$ (Eq. (A15)) in the region where $|S_1|+ |S_2|\le 1$. We also need to show $\mathcal {M}_S$ is a minimum in the region where $|S_1|+ |S_2|> 1$. By Eq. (A7), the condition $|S_1|+ |S_2|> 1$ is equivalent to ${1}/{u_1^c}+{1}/{u_2^c}> L$. Therefore we have the following constraints

A19 $$\begin{aligned} \frac{1}{u_1^c}+\frac{1}{u_2^c}&> 1, \nonumber \\ u_i^c&\ge 1,\quad \text {for}\quad i=1,2. \end{aligned}$$

As already shown in “Appendix A.1” (see Eq. (A11)), when $|S_1|+ |S_2|> 1$ the function $\mathcal {E}({u}_{1}^c,{u}_{2}^c)$ (Eq. (A6)) can be rewritten as

A20 $$\begin{aligned} \mathcal {E}(u_1^c,u_2^c)&=\text {ln}(u_1^c)+\text {ln}(u_2^c) + \frac{1}{2}\gamma _{11} (u_1^c+u_1^c)+ \gamma _{12} u_1^c u_2^c \left( \frac{1}{u_1} + \frac{1}{u_2} -1\right) . \end{aligned}$$

To show that $\mathcal {M}_S$ (Eq. (A18)) is a minimum on the region of the domain given by Eq. (A19), we perform a Taylor expansion of $\mathcal {E}(u_1^c,u_2^c)$ (Eq. (A20)) around $\mathcal {M}_S$ within this region. Since the slope of the tangent line to the curve $1/u_1^c+1/u_2^c = 1$ at the point $\mathcal {M}_S$ is $-1$, we choose two arbitrary constants, $\epsilon$ and $\delta$, such that $\epsilon + \delta \le 0$. The Taylor expansion is then

A21 $$\begin{aligned} \mathcal {E}(2+\epsilon ,2+\delta )&\approx \mathcal {E}(2,2)+ \partial _{u_1^c} \mathcal {E}(2,2) \epsilon + \partial _{u_2^c}\mathcal {E}(2,2)\delta \nonumber \\&=\mathcal {E}(2,2)+\frac{1}{2}(1+ \gamma _{11}-2\gamma _{12})(\epsilon +\delta ) \nonumber \\&\ge \mathcal {E}(2,2), \end{aligned}$$

if $\gamma _{11} < 2\gamma _{12}-1$. Therefore, $\mathcal {M}_S$ (Eq. (A18)) is a local minimum of $\mathcal {E}(u_1^c,u_2^c)$ (Eq. (A15)) when $-1<\gamma _{11} < 2\gamma _{12}-1$. We recall that if $(u_1^c,u_2^c)=\mathcal {M}_S$ then the functions $u_i(x)$ (Eq. (A2)) that locally minimize the energy $E[u_1 ,u_2 ]$ (Eq. (A5)) correspond to the class of functions $\mathcal {S}_{S}^{2,2}$ defined in Eq. (A14).

Next we look for other local minima within the region of the domain given by Eq. (A19). A direct calculation using partial derivatives shows that there are no local minima of $\mathcal {E}(u_1^c,u_2^c)$ in the interior of this region. We now verify whether local minima occur on the boundaries. On the part of the boundary given by $u_i^c =1$, for $i=1,2$, there is a local minimum at

A22 $$\begin{aligned} \mathcal {M}_H=(u_{1H}^c, u_{2H}^c):=\left( 1,1\right) . \end{aligned}$$

This is also a local minimum of the region defined by Eq. (A19) when $\gamma _{11}>-2$. This can be shown by performing a Taylor expansion of $\mathcal {E}(u_1^c,u_2^c)$ (Eq. (A20)) about the point $\mathcal {M}_H$, to give

$$\begin{aligned} \mathcal {E}(1+\epsilon ,1+\delta )&\approx \mathcal {E}(1,1)+ \partial _{u_1^c} \mathcal {E}(1,1) \epsilon + \partial _{u_2^c} \mathcal {E}(1,1)\delta \\&= \mathcal {E}(1,1)+ \left( 1+\frac{1}{2}\gamma _{11}\right) (\epsilon +\delta ) \\&\ge \mathcal {E}(1,1), \end{aligned}$$

where the inequality uses $\gamma _{1 1}>-2$, $\epsilon \ge 0$ and $\delta \ge 0$. Note that $\epsilon$ and $\delta$ are chosen to be non-negative so that we remain in the $u_i^c \ge 1$ region (Fig. 2). We recall that if $(u_1^c,u_2^c)=\mathcal {M}_H$ then the functions $u_i(x)$ (Eq. (A2)) that locally minimize the energy $E[u_1 ,u_2 ]$ (Eq. (A5)) correspond to $\mathcal {S}_H$, the homogeneous steady state.

Notice also that on the boundary $$u_{1}^c=1$$, $$\mathcal {E}(u_1^c,u_2^c)$$ (Eq. (A20)) decreases as $$u_2^c\rightarrow \infty $$ and, analogously, on the boundary $$u_{2}^c=1$$, $$\mathcal {E}(u_1^c,u_2^c)$$ (Eq. (A20)) decreases as $$u_1^c\rightarrow \infty $$. Therefore, by keeping $$u_{i}^c=1$$ fixed, for $$i=1,2$$, $$\mathcal {E}(u_1^c,u_2^c)\rightarrow -\infty $$ as $$u_{j}^c \rightarrow \infty $$, for $$ j\ne i $$. As we approach this limit, the function $$u_j(x) $$ (Eq. (A2)) becomes an arbitrarily high function with an arbitrarily narrow support, while $$u_i(x) $$ (Eq. (A2)), for $$i\ne j$$, remains at finite height. We denote the limit of these solutions by $$\mathcal {S}_S^{1,\infty }$$.

In conclusion:

- If $\gamma _{11}> 2\gamma _{12}-1$, then the energy $E(u_1^c,u_2^c)$ (Eq. (A2)) has the following local minima: $\mathcal {S}_H$, $\mathcal {S}_S^{\infty ,\infty }$ and $\mathcal {S}_S^{1,\infty }$.
- If $-1<\gamma _{11}<2\gamma _{12}-1$, the energy $E(u_1^c,u_2^c)$ (Eq. (A2)) has the following local minima: $\mathcal {S}_H$, $\mathcal {S}_S^{\infty ,\infty }$, $\mathcal {S}_S^{1,\infty }$ and $\mathcal {S}_S^{2,2}$.
- If $-2<\gamma _{11}<-1$, the energy $E(u_1^c,u_2^c)$ (Eq. (A2)) has the following local minima: $\mathcal {S}_H$, $\mathcal {S}_S^{\infty ,\infty }$ and $\mathcal {S}_S^{1,\infty }$.
- If $\gamma _{11}<-2$, the energy $E(u_1^c,u_2^c)$ (Eq. (A2)) has the following local minima: $\mathcal {S}_S^{\infty ,\infty }$ and $\mathcal {S}_S^{1,\infty }$.

Furthermore, linear stability analysis (Eq. (14)) suggests that when $\alpha$ tends to zero, the homogeneous steady state is stable if $\gamma _{11}> \gamma _{12}-1$. This gives rise to the diagram of analytically-predicted steady states given by the red and black lines in Fig. 8.

## Appendix B: Details of calculations from Section 5.2

Here, we analyze the solutions to the system $\left\{ \det (A_1^{(2)})=0, \det (A_2^{(2)})=0\right\}$, where $A_1^{(2)}$ and $A_2^{(2)}$ are given in Eqs. (58) and (61), respectively. We write the system $\left\{ \det (A_1^{(2)})=0, \det (A_2^{(2)})=0\right\}$ in full as

B23 $$\begin{aligned} 0&=(D_1+\gamma _{11}u_1 )(D_2+\gamma _{22}u_2 )-\gamma _{12}\gamma _{21}u_1 u_2 , \end{aligned}$$

B24 $$\begin{aligned} 0&=\gamma _{12} u_1 \left( \gamma _{11} \left( D_2+\gamma _{22} u_2\right) -\gamma _{12}\gamma _{21} u_2\right) \nonumber \\&\quad -\left( \gamma _{22} \left( D_1+\gamma _{11} u_1\right) -\gamma _{12}\gamma _{21} u_1\right) \left( D_1+\gamma _{11} u_1\right) , \end{aligned}$$

By subtracting Eq. (B24) from Eq. (B23), we obtain the following linear equation in $u_2$

B25 $$\begin{aligned}&\gamma _{11} D_2 u_1-\gamma _{11} \gamma _{12} D_2 u_1-\gamma _{12} \gamma _{21} D_1 u_1+\gamma _{22} \left( D_1+\gamma _{11} u_1\right) {}^2 \nonumber \\&\quad +D_1 D_2-\gamma _{11} \gamma _{12}\gamma _{21} u_1^2\nonumber \\&\quad + u_2 (\gamma _{22} D_1+\left( \gamma _{12}-1\right) \left( \gamma _{12} \gamma _{21}-\gamma _{11} \gamma _{22}\right) u_1)=0. \end{aligned}$$

By using Eq. (B25) to find $u_2$ in terms of $u_1$ and then substituting this into Eq. (B23), we obtain the following cubic equation in $u_1$

B26 $$\begin{aligned}&\gamma _{22}^2 D_1^3 - D_1 u_1 \left( \gamma _{21} \gamma _{12}^2 D_2+\gamma _{22} \left( 2 \gamma _{12} \gamma _{21}-3 \gamma _{11} \gamma _{22}\right) D_1\right) \nonumber \\&\quad + u_2^2 D_1 \left( \gamma _{12} \gamma _{21}-3 \gamma _{11} \gamma _{22}\right) \left( \gamma _{12} \gamma _{21}-\gamma _{11} \gamma _{22}\right) \nonumber \\&\quad +u_1^3\gamma _{11} \left( \gamma _{12} \gamma _{21}-\gamma _{11} \gamma _{22}\right) {}^2 =0. \end{aligned}$$

Since Equation (B26) has at most three roots, System (B23)–(B24) has at most three solutions.
